# Potential Role of Dietary Phenolic Compounds in the Prevention and Treatment of Rheumatoid Arthritis: Current Reports

**DOI:** 10.3390/ph17050590

**Published:** 2024-05-06

**Authors:** Ana C. Gonçalves, Sofia Rodrigues, Rafael Fonseca, Luís R. Silva

**Affiliations:** 1 CICS-UBI—Health Sciences Research Center, University of Beira Interior, 6201-001 Covilhã, Portugal; anacarolinagoncalves@sapo.pt; 2 CIBIT—Coimbra Institute for Biomedical Imaging and Translational Research, University of Coimbra, 3000-548 Coimbra, Portugal; 3 SPRINT Sport Physical Activity and Health Research & Innovation Center, Instituto Politécnico da Guarda, 6300-559 Guarda, Portugal; 4 Health Superior School, Polytechnic Institute of Viseu, 3500-843 Viseu, Portugal; rodriguessofia944@gmail.com; 5 Faculty of Medicine, University of Lisbon, 1649-028 Lisbon, Portugal; rafaelfonseca1@edu.ulisboa.pt; 6 CERES, Department of Chemical Engineering, University of Coimbra, 3030-790 Coimbra, Portugal

**Keywords:** anti-inflammatory activity, phenolic compounds, rheumatoid arthritis, diet, quality of life

## Abstract

Rheumatoid arthritis (RA) is a complex illness with both hereditary and environmental components. Globally, in 2019, 18 million people had RA. RA is characterized by persistent inflammation of the synovial membrane that lines the joints, cartilage loss, and bone erosion. Phenolic molecules are the most prevalent secondary metabolites in plants, with a diverse spectrum of biological actions that benefit functional meals and nutraceuticals. These compounds have received a lot of attention recently because they have antioxidant, anti-inflammatory, immunomodulatory, and anti-rheumatoid activity by modulating tumor necrosis factor, mitogen-activated protein kinase, nuclear factor kappa-light-chain-enhancer of activated B cells, and c-Jun N-terminal kinases, as well as other preventative properties. This article discusses dietary polyphenols, their pharmacological properties, and innovative delivery technologies for the treatment of RA, with a focus on their possible biological activities. Nonetheless, commercialization of polyphenols may be achievable only after confirming their safety profile and completing successful clinical trials.

## 1. Introduction

There exist more than 100 conditions of arthritis known, and among them, rheumatoid arthritis (RA) is one of the most common forms observed in the elderly population [1]. RA affects 0.1–2.0% of the population worldwide, being three times more common in the female gender. The condition can begin at any age, although around 80% of all individuals develop the disease between 35 and 50 years old. Their etiology remains poorly understood, and despite recent therapeutic advances, there is no known cure [2].

RA is a systemic autoimmune, chronic, heterogeneous, and inflammatory disease in which the immune system mistakenly attacks healthy cells in the body, reducing quality of life and shortening its duration [3,4]. RA is a complex process involving numerous inflammatory mediators and is characterized primarily by aggressive synovial hyperplasia, synovitis, progressive cartilage degeneration, and bone erosion with painful swelling of small joints, fatigue, prolonged stiffness, and fever produced by immune responses and particular innate inflammatory processes [4,5]. Although RA affects multiple body systems, the joints of the hands, wrists, feet, ankles, knees, shoulders, and elbows are most often affected [6]. The lining of joints becomes inflamed in RA joints, causing joint tissue destruction. Long-term or chronic discomfort, unsteadiness (loss of balance), and deformity (misshapenness) can result from this tissue injury. RA can also affect other tissues and organs, including the lungs, heart, and eyes [1,7].

Although RA changes have been extensively investigated, there is a still lack of effective drugs. During RA, there are many pro-inflammatory cytokines, such as interleukin (IL)-1, IL-6, IL-17, and TNF-*α*, and chemokines released to the synovial space [4]. Therefore, it is urgent to find effective drugs for RA by inhibiting the production and release of pro-inflammatory cytokines. To date, non-steroidal anti-inflammatory agents and disease-modifying anti-rheumatic drugs, like immunosuppressants and monoclonal antibodies (e.g., rituximab and tocilizumab) combined with methotrexate, have been largely used to attenuate RA; however, most of them are expensive, rarely effective or tolerable, and lead to several unwanted effects, including allergy and infections, gastrointestinal problems, fluid retention, renal dysfunction and systemic vasculitis, cytopenia, lymphopenia, neutropenia, and the elevation of transaminase and cholesterol [7,8,9,10,11,12,13]. Among the new and most promising availability strategies, special attention has been given to bioactive molecules largely found in nature, namely, polyphenols, since their chemical structure with catechol methoxy and pyrogallol groups confer them notable health benefits, like antioxidant, anti-inflammatory, anti-bacterial, and antiproliferative activities, as well as the capacity to prevent neurological and cardiovascular pathologies, promote gastrointestinal health and vision without, it is believed, side-effects, and enhance wound healing [14,15,16,17,18,19,20,21,22,23]. In addition, polyphenols are also easier to obtain, water-soluble, and more economical than chemical drugs, and have already shown effectiveness in inhibiting JAK proteins and JAK/STAT signaling pathways [24,25,26,27,28]. Particularly, curcumin displayed the capability to relieve rheumatoid arthritis progression by suppressing inflammatory responses, synovial hyperplasia, and protein expression levels of phosphorylated JAK2 and STAT3 in mice with the collagen-induced arthritis model that were treated at a dose of 100 µM curcumin/day for almost three months [29], while in docking studies, salvianolic acid C exhibited a great binding affinity for JAK (10.7 kcal/mol) [24]. In addition, phenolic-rich extracts of sweet cherries have already displayed a notable ability to scavenge cellular nitric oxide species and diminish inducible nitric oxide synthase (iNOS) and cyclooxygenase (COX)-2 expression [30], while tea polyphenols showed the capacity to enhance the expression of growth factor VEGF-A, which, in turn, promotes vascular endothelial cell division, induces angiogenesis, and improves wound morphology, restoring the type III/I collagen ratio during wound healing in mice model [23].

Furthermore, in addition to acting as nutraceuticals and functional foods, evidence shows that those polyphenols can also present many industrial applications, being used for water treatment and as natural food additives, natural dyes for textiles, smart food packaging and edible biofilms, anti-browning and food improvement agents, food enrichment and colorant, anti-aging creams and sunscreens, and hair products and nail remedies [31,32].

Keeping these facts in mind, the main goal of this work is to review and discuss the dietary polyphenols commonly found in the human diet, their rich sources in preventing and/or attenuating RA, and the most advanced strategies and also suggest future research.

## 2. Data Collection

The data collection was performed by searching, from the time of their establishment to 24 March 2024, scientific databases including National Center for Biotechnology Information (NCBI), Google Scholar, PubMed, ResearchGate, Science Direct, Scopus, SpringerLink, Web of Science, and trusted abstracts. The free terms, keywords, and MeSH terms used were polyphenols, phenolics, total phenolic content, phenolic-rich sources, antioxidant effects, anti-inflammatory properties, health benefits, and rheumatoid arthritis combined with AND, OR, or NOT operators. During the literature review, there were no restrictions on the author(s) or type of publication. In total, 369 papers were cited in this review.

## 3. Bioavailability and Metabolism of Polyphenols

After daily intake, polyphenols suffer many modifications along the gastrointestinal tract, which greatly influence their nutritional value and consequent health benefits. Therefore, exploring their bioavailability and metabolism is crucial to reveal the full potential of these compounds, of which the majority are considered non-toxic and safe for people [33,34].

Nowadays, it is well-established that polyphenolics are subject to extensive metabolization after consumption, and hence, it is not surprising that absorption is higher than expected, and some of them can be almost totally assimilated. In general, the maximum plasma concentration of polyphenolics is reached 1.5–2 h after ingestion, and excretion rates around 30%, varying between 12 h (flavanols) and 24 h (anthocyanins and hydroxycinnamics) [35,36].

Within different subclasses, isoflavones seem to exhibit the highest absorption rates (33.0–100.0%), followed by hydroxycinnamics (8.0–72.0%), anthocyanins (2.40–55.0%), flavonols and flavones (12.0–41.0%), flavanones, (11.0–16.0%), lignans (2.70–12.20%), and flavan-3-ols (2.0–8.0%) [34,37].

As far as we know, both the bioavailability and metabolism of polyphenol parameters differ significantly between different polyphenol compounds, and sometimes, the most dominant in our diet are not necessarily those that show the highest concentrations of active derivatives in tissues and organs. Indeed, molecular weight, glycosylation and/or acylation and/or polymerization degrees, conjugations, and/or combinations with other molecules impact bioavailability [38]. In addition, different pH values also influence these characteristics, as well as solubility, digestibility, the capacity to cross cell membranes, cooking, agronomic practices, food maturity index, and if they are consumed alone, and/or after an overnight fasting and/or accompanied with other foods or alone [39]. It is also important to take into account individual genetics, the existence of food intolerances, gender, age, sex, diet, lifestyle, and physiological and/or pathological states, which also interfere with different activities of gut microbiota, enterocytes, related enzymes, and biliary acids, also impact the bioavailability, metabolism and consequent biological effects of these phytochemicals [38].

**Figure 1:**
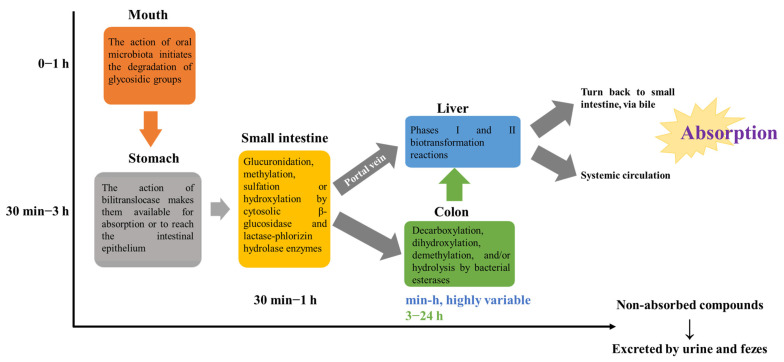
Summary of polyphenol absorption, distribution, metabolism, and excretion in different segments of the gastrointestinal tract (adapted Olivero-David and co-workers [40] and Ray et al. [41].

Figure 1 summarizes the main steps involved in the absorption, metabolism, distribution, and excretion of polyphenols.

As expected, and before reaching systemic circulation, there is in the mouth that the degradation of polyphenolics initiates. Here, saliva enzymes remove glycoside groups, and then the compounds reach the stomach, where simpler aglycones, like isoflavones and phenolic acids, such as gallic acid, can be readily absorbed by bilitranslocase (bioaccessibility) or reach intestinal epithelium by passive diffusion thanks to their high lipophilicity or by using active sodium-dependent glucose cotransporters, including glycosides, polymers, and esters, becoming available for absorption [42]. On the other hand, the most complex polyphenols go to the small intestine. Here, they suffer glucuronidation, methylation, sulfation, or hydroxylation by cytosolic β-glucosidase and lactase-phlorizin hydrolase enzymes [41]. Next, simpler compounds reach the liver via the portal vein and the non-absorbed ones go to the colon to be transformed into simpler units, undergoing decarboxylation, dihydroxylation, demethylation, and/or hydrolysis thanks to the activity of bacterial esterases, including α-rhamnosidases, to suffer a new attempt at absorption by the liver [43]. Indeed, evidence reports that most polyphenols are only absorbed after being metabolized in the colon (90.0–95.0%) [44]. This event also leads to pH diminution by forming short-chain fatty acids, which, in turn, creates favorable conditions for the proliferation of Actinobacteria, Lactobacilli, and Bifidobacteria probiotic bacteria, hence exerting protective effects against digestive and gastrointestinal disorders, allergies, eczema, and others [38].

Therefore, microbiota causes hydrolysis of glycosidic and ester bonds and cleavage of aglycones into smaller molecules, while hepatocytes produce mainly phase I and II metabolites (conjugates) from intestinal metabolites such as aglycones, phenolic acids, and simple phenols [45]. Phase II also occurs in the small intestine and kidneys [46]. In addition, the non-absorbed polyphenols in the liver can go again, via bile, to the small intestine to be metabolized again [47]. The non-absorbed polyphenols are excreted in urine and feces [47].

Even so, to enhance the absorption, metabolism, and stability of polyphenolics, several strategies have been conducted to improve these topics. Among the most promising ones, nano-encapsulation with biopolymers, chitosan, pectin, whey protein, and liposomal micelle have been hot topics in scientific communities and medicine, pharmaceutical, and other related industries [48,49].

## 4. Major Sources of Dietary Polyphenols

**Figure 2:**
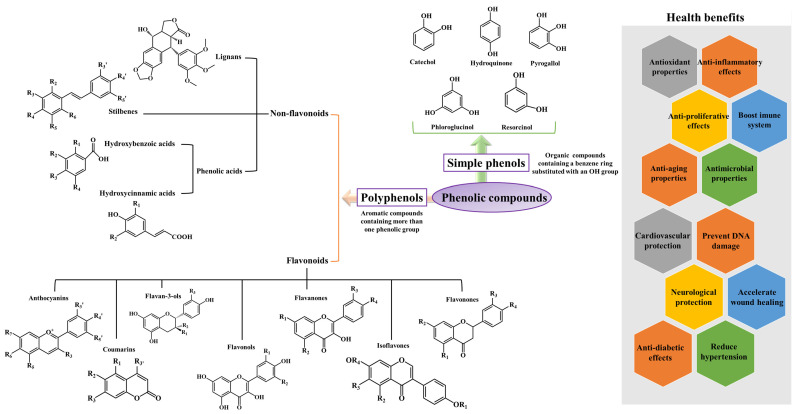
Main dietary polyphenols and simple phenols including their most notable health-benefits (OH: hydroxyl group) (adapted from Mamari [50], Gonçalves et al. [51], and Matsumura et al. [52]).

Nowadays, the search for more effective, safer, and cheaper bioactive compounds than chemical drugs assumes great importance. Indeed, because most chemical drugs possess several unwanted effects and are little active, many people prefer to use natural molecules [33]. Among the most promising molecules, phenolic compounds, including simple phenols (e.g., catechol, pyrogallol, phloroglucinol, and resorcinol) and polyphenols (e.g., coumarins, anthocyanins, lignans, and phenolic acids) seem to be promising therapeutic and/or adjuvant approaches (Figure 2) [50,51,52]. Simple phenolics have a low molecular weight, and most of them (except resorcinol) are rapidly absorbed by the human skin, while polyphenols are largely widespread and ubiquitous in nature, being largely found in several types of algae, beans, fruits, bark, herbs and spices, legumes, leaves, nuts, whole grains, oilseeds, and roots (Table 1) [53,54]. In a general way, polyphenols can occur in free forms or conjugated with sugar moieties, acids, and other biomolecules that are soluble or insoluble in water, and, of course, these substitutions can interfere with the biological potential of these compounds [55]. Indeed, depending on the number and spatial position of hydroxyl groups, they can confer polyphenols with a great capacity to act as electron and hydrogen donors [56]. In addition, the CH=CH–COOH group and the double bond found in hydroxycinnamic acids make them more effective in reducing oxidative stress than hydroxybenzoics [57]. On the other hand, the presence of an *o*-diphenolic group and catechol hydroxyl groups, a double bond, and the conjugation between the double bond and the oxo group on the C ring enhance the antioxidant properties of flavonoids [58]. It is also important to note the obstruction of the *O*-methylation at the catechol group can diminish the biological potential of these phytochemicals [59].

Noticeably, stilbenes are predominant in grapevines, peanuts, and sorghums (amounts of 0.16–0.77 mg per 100 g, 1.8–787.3 µg per 100 g, and up 0.01 mg per 100 g, respectively), showing total polyphenol compounds (TPCs) of 9.3–75.3, 94.4–228.4, and 100–2300 mg GAE per 100 g fresh weight (fw), respectively [60,61,62,63]. Lignans are highly present in flaxseeds (amounts of 9 to 30 mg per g, being 75 to 800 times higher than in cereals, fruits, legumes, and vegetables), revealing TPC scores around 3000 mg per 100 g [64,65]; in addition, flaxseeds also present considerable quantities of phenolic acids (800–1000 mg per 100 g) [66].

Focusing on phenolic acids, hydroxybenzoic derivatives are mainly found in black radish, onions, tea, and wine [67,68,69,70]. Particularly, gallic acid is found in considerable amounts in guava [4.21 mg/100 g dried weight (dw)], white mulberry (7.33–23.90 mg/100 g dw), mango (94.6–98.7 mg/100 g dw), avocado (0.56–2.54 mg/100 g dw), raspberry (0.73–3.75 mg/100 g dw), grape skins and seeds (3 and 10 mg/100 g dw, respectively), and blackcurrant leaves (17.98–22.85 mg/100 g dw) [71]. On the other hand, hydroxycinnamics are largely present in cereals, fruits, vegetables, tea, wine, and coffee [71,72,73,74]. Particularly, plums and sweet and tart cherries are richer in 3-*O*-caffeolquinic acid (amounts of 8.8–77.1, 7.3–62.8, and 8.2–53.6 mg/100 g fw, respectively) [67,75,76]. On the other hand, 5-*O*-caffeolquinic acid is largely present in apples and pears (both around 0.2 mg/100 g fw), while 3-feruloyquinic acid is mostly found in plums and apricots (quantities around 1.3 and 0.7 mg/100 g fw, respectively), caffeoylquinic acids in black currants (amounts between 4.5–5.2 mg/100 g fw), and *p*-coumaroylquinic derivatives in blueberries (186.0–208.0 mg/100 g fw) [67,77]. Regarding vegetables, 3-*O*-caffeolquinic acid is predominant in red cabbage (1.9–11.0 mg/100 g fw), Brussel sprouts (7.0–12.0 mg/100 g fw), broccoli (5.8 mg/100 g fw), and kale stalk leaves (0.61–7110.7 mg/100 g fw). Kale stalk leaves also present notable amounts of feruloyl glucose acid derivative (31.71 mg/kg) [67]. On the other hand, eggplant is richer in 4-*O*-caffeolquinic acid (57,563.2 mg/100 g fw) [67].

Focusing on anthocyanins, they are widely found in red and purple fruits and vegetables and are considered as being mainly responsible for their vibrant colors and health-promoting properties. Specifically, cyanidin 3-*O*-rutinoside is mainly found in sweet cherries, while cyanidin 3-*O*-glucosyl-rutinoside is the predominant anthocyanin in sour cherries [75,78]. Regarding TPC values, sour cherries present higher values than sweet cherries (275.3–652.27 versus 72.9–493.6 mg gallic acid equivalent (GAE) per 100 g fw for sweet cherries) [78,79,80]. On the other hand, the TPC value of blueberries fluctuates between 2.7 and 585.3 mg GAE per 100 g fw, being richer in peonidin derivatives, whereas for black elderberries, the TPC value is around 537.9 mg GAE per 100 g fw, and cyanidin 3-*O*-sambubioside is the main anthocyanin [77,81,82,83]. Blackberries also present considerable amounts of TPC (292.2–446.4 mg GAE per 100 g fw), where cyanidin 3-*O*-glucoside is the major anthocyanin [84,85]. For strawberries, the TPC value varies from 36.5 to 116.3 mg GAE per 100 g fw, where the most abundant is pelargonidin 3-*O*-glucoside [86,87,88], while for grapevines (TPC 9.3–75.3 mg per 100 g fw), malvidin 3-*O*-glucoside is the predominant anthocyanin [63,89,90]. Additionally, mulberries and chokeberries also present considerable amounts of TPCs (424–485 and 1022.4–1705.9 mg GAE per 100 g fw, respectively), where cyanidin 3-*O*-sophoroside and cyanidin 3-*O*-galactoside are the predominant ones, respectively [91,92,93], whereas black currants are richer in delphinidin 3-*O*-rutinoside and present TPC values fluctuating from 1930.0 to 3410.0 mg GAE per 100 g fw [94,95]. Red cabbage also presents considerable TPC scores (115.31 mg per 100 g fw), where the most prevalent anthocyanin is cyanidin 3-*O*-diglucoside-5-*O*-glucoside [96,97].

Isoflavones (e.g., genistein and daidzein) are abundant in soybeans (total isoflavones and TPC values of 80.7–213.6 mg per 100 g and 87.2–216.3 mg per 100 g fw, respectively); within isoflavones, genistein is the most found, followed by daidzein (21.4–78.3 and 15.0–67.4 mg per 100 g, respectively) [98]. Regarding coumarins, marmelosin is found in high quantities in Aegle quince fruits (290 g per 100 g for TPC values varying from 905.0 to 4900.0 mg GAE per 100 g) [99,100], while esculin is predominantly found in horse-chestnut bark, accounting for around 7.9% of their total composition (33–50 mg/100 g) [101,102].

Regarding flavan-3-ols, the presence of (+)-catechin was reported in peaches (30–150 mg/100 g fw), apricots (2.1–3.2 mg/100 g fw), apples (0.4–2.1 mg/100 g fw), and green tea (50–100 mg in 250 mL), whereas (−)-epicatechin was reported in apricots (2.01–6.50 mg/100 g fw), sour cherries (1.1–9.6 mg/100 g), apples (3.1–7.8 mg/100 g fw), and cocoa (34.65–43.27 mg/g fw) [103,104,105,106,107,108,109,110,111]. On the other hand, epigallocatechin gallate is the major flavan-3-ol found in green tea (68–69%), followed by (−)-epigallocatechin (15–18%), (−)-epicatechin (5–6%), and epicatechin (2–5%) [112,113,114,115].

Flavonols, especially kaempferol derivatives, are largely present in onion leaves (amounts around 83.2 mg/100 g), papaya shoots (amounts around 45.3 mg/100 g fw), pumpkins (amounts around 37.1 mg/100 g), carrots (amounts around 14.0 mg/100 g), and black tea (amounts around 11.8 mg/100 g) [116,117,118,119]. On the other hand, *Mentha pulegium* L. is rich in quercetin aglycone (8.48 mg/100 g) [120,121], while dill (79.0 mg/100 g fw), onions (45.0 mg/100 g fw), oregano (42.0 mg/100 g fw), spinach (27.2 mg/100 g fw), and cranberries (25.0 mg/100 g fw) are rich in quercetin derivatives [122]. Myricetin derivatives are largely found in cranberries (6600 mg/100 g fw), dock (5700 mg/100 g fw), broad beans (2600 mg/100 g fw), rutabagas (2100 mg/100 g fw), blueberries (1300 mg/100 g fw), peppers (1200 mg/100 g fw), and blackberries (700 mg/100 g fw) [123]. Finally, fisetin derivatives are principally reported in strawberries (16 mg/100 g fw), apples (2.7 mg/100 g fw), persimmon (1.1 mg/100 g fw), lotus root (0.6 mg/100 g fw), onions (0.5 mg/100 g fw), grapes (0.4 mg/100 g fw), kiwi (0.2 mg/100 g fw), peaches (0.06 mg/100 g fw), and cucumber (0.01 mg/100 g fw) [124].

Relative to flavanone compounds, higher levels of hesperetin are found in dried peppermint (480.85 mg/100 g), honeybush tea (11.82 mg/100 g), onions (0.02 mg/100 g fw), clementine (39.9 mg/100 mL), sweet oranges (28.6 mg/100 mL), tangerines (24.3 mg/100 mL), lemons (20.5 mg/100 mL), limes (1.77 mg/100 mL), grapefruit pure juice (0.92 mg/100 g), and mandarins (0.15 mg/100 mL) [125,126]. On the other hand, naringin is mainly present in grapefruit pure juice (1.56 mg/100 mL), red wine (0.07 mg/100 mL), Mexican oregano (372 mg//100 g), rosemary (55.1 mg/100 g), sweet oranges (11.22 mg/100 g fw), tomatoes (0.96–3.84 mg/100 g fw), limes (3.40 mg/100 g fw), lemons (0.55 mg/100 g fw), and almonds (0.09 mg/100 g) [126,127]. Eriodictyol is present in Yerba Santa (23–795 mg/100 g) [128].

Focusing on flavones, apigenin is the most predominant compound found in nature, being largely reported in celery seeds (78.65 mg/100 g), spinach (62.0 mg/100 g), parsley (45.04 mg/100 g), marjoram (4.40 mg/100 g), oregano (3.50 mg/100 g), sage (2.40 mg/100 g), extra-virgin oil (1.17 mg/100 g), rosemary (0.55 mg/100 g), chamomile (3–5 mg/100 g), and pistachios (0.03 mg/100 g) [127].

Regarding flavonones, hesperidin derivatives are also predominant in citrus-based foods (0.026 mg/100 mL) [129,130], while honey and propolis present higher levels of pinocembrin (1.60–1.85 mg/100 g and 0.079–0.166 mg/100 mL, respectively) [131,132,133], and *Glycyrrhiza glabra* L. plant leaves and roots of liquiritin (1821 mg/100 g and 3336 mg/100 g, respectively) [132,134].

Focusing on ellagitannins, they were found to be only present in berries from the Rosaceae family, including strawberries and raspberries, rose hip, cloudberries, and sea buckthorn (amounts varying from 21.7 to 83.2 mg/100 g fw) [135]. Particularly, punicalagin is predominantly found in pomegranate peels (2803–10,414 mg/100 g) [136].

Regarding curcuminoid compounds, turmeric presents higher amounts of curcumin (877–1648 mg/100 g), 2-demethoxycurcumin (364–760 mg/100 g), and bisdemethoxycurcumin (276–431 mg/100 g fw) [137].

Of course, quantity values are greatly influenced by genotype, origin, climate, time of harvest, agricultural and processing techniques, and so on [138].

**Table 1:** Main polyphenols found in nature and their phenolic-rich sources.

Polyphenols	Sources	References
Non-flavonoids		
Lignans	Flaxseeds	[139]
Phenolic acids Hydroxybenzoic acid derivatives Hydroxycinnamic acid derivatives	Black radish, onions, tea, guava, white mulberry, mango, avocado, raspberry, grape skins and seeds, and blackcurrant leaves Cereals, coffee, fruits, tea, vegetables, and wine	[67,68,69,70,71]
Stilbenes	Grapevines, peanuts and sorghums	[140]
Flavonoids		
Anthocyanins Cyanidin derivatives Delphinidin derivatives Pelargonidin derivatives Peonidin derivatives Petunidin derivatives Malvidin derivatives	Sweet and sour cherries, mulberries, black elderberries, chokeberries, and red cabbage Eggplant, roselle, maqui berries, and black currants Raspberries and strawberries Cranberries, blueberries, and plums Chokeberries Acerola, blackberries, and grapevines	[75,77,88,94,141,142,143,144,145,146,147,148,149,150,151,152]
Coumarins Umbelliferone Esculin	Aegle marmelo Horse-chestnut bark	[100,101,153]
Flavan-3-ols (+)-Catechin (−)-Epicatechin Epigallocatechin gallate	Peaches, apricots, apples, and green tea Apricots, sour cherries, apples, cholate, and cocoa Green tea	[67,103,154,155,156]
Flavonols Quercetin Kaempferol derivatives Quercetin derivatives Myricetin derivatives Fisetin derivatives	*Mentha pulegium* L. Onion leaves, papaya shoots, pumpkins, carrots, and black tea Dill, onions, spinach, and cranberries Cranberries, dock, broad beans, rutabagas, blueberries, peppers, and blackberries Strawberries, apples, persimmons, grapes, onions, and cucumbers	[116,118,119,120,121,122,123,157,158]
Flavanones Hesperetin Naringin Eriodictyol	Dried peppermint, honeybush tea, onions, clementine, sweet orange, tangerine, lemon, lime, grapefruit pure juice, and mandarins Grapefruit pure juice, red wine, Mexican oregano, rosemary, sweet oranges, tomatoes, limes, lemons, and almonds Yerba Santa	[127,159,160,161,162]
Flavonones Hesperidin Liquiritin Pinocembrin	Citrus fruits *Glycyrrhiza glabra* L. leaves and roots Honey and propolis	[163,164,165,166]
Flavones Apigenin	Celery seeds, spinaches, parsley, marjoram, oregano, sage, extra virgin oil, rosemary, chamomile, and pistachio	[127]
Isoflavones	Soybeans	[98,167,168,169,170,171]
Ellagitannins Punicalagin	Strawberries and raspberries, rose hip, cloudberries, and sea buckthorn Pomegranate	[135] [136]
Curcuminoids Curcumin	Turmeric	[137]

## 5. Pharmacological Properties of Dietary Polyphenols

Based on deleterious effects caused by oxidative stress and exacerbated inflammatory responses, it is not surprising that these natural molecules, which are composed of many hydroxyl groups, can easily neutralize radical species or break chains, by giving an electron or hydrogen atom, and interfere with inflammatory pathways, thus contributing to increase immunity defenses and promoting a healthy status. In addition, evidence also shows that they are effective in acting as anti-aging, anti-microbial, and antiproliferative agents and possess abilities to increase insulin sensitivity and digestion, lower blood sugar levels, and ameliorate brain and heart functions.

**Figure 3:**
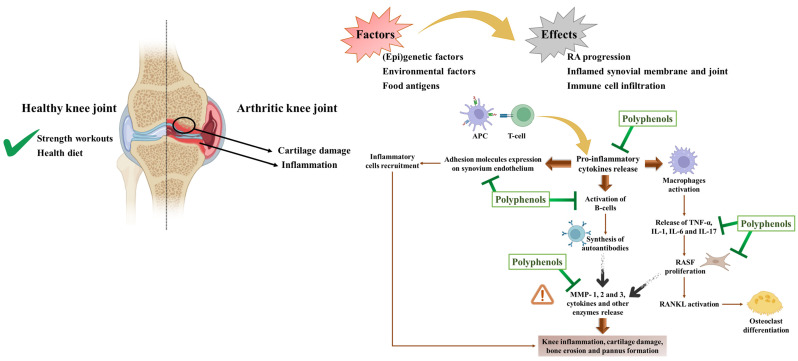
Effects of polyphenols against rheumatoid arthritis (RA: rheumatoid arthritis; APC: antigen-presenting cell; IL: interleukin; TNF-*α*: tumor necrosis factor alpha; MMP: matrix metalloproteinases; RASF: rheumatoid arthritis synovial fibroblasts; RANKL; receptor activator of nuclear factor kappa-Β ligand) [Portions of Figure 3 were drawn using images from BioRender.com (https://biorender.com/) (accessed on 8 March 2024)] (adapted from Weyand and Goronzy [178] and Long et al. [6]).

As expected, their benefits are closely linked to their chemical structure and quantities. Indeed, several studies already reported that, in most cases, an increment in phenolics enhances biological potential [65,75,79,172,173,174,175,176,177]. Therefore, it is not surprising that their interest is increasing worldwide, especially in preventing and/or attenuating several diseases, especially those without medical cures, like AR (Figure 3). In this disease, there is a verified network of inflammatory components, degrading enzymes, angiogenic molecules, and cells. Altogether, this originates immune deregulation, which, without surprise, is not only associated with exacerbated inflammatory responses but also with oxidative stress, affecting particularly the nuclear factor erythroid 2–related factor 2 (Nrf2) pathway (discussed below). Phenolics have shown potential to counteract oxidative stress levels and downregulate exacerbated inflammatory responses, and thus, their use as adjuvant therapy and/or combined with chemical pharmaceutics can be very useful. Similar approaches have been applied in cancer treatment. In fact, in 2019, of the 247 anticancer drugs available, 200 were from natural products while 38 were synthetic drugs and nine were vaccines [33].

### 5.1. Antioxidant Capacity

Reactive oxygen and nitrogen species, namely, superoxide and nitric oxide radicals, play a vital part in the human body, which are signaling molecules that interfere with immune responses and signal transduction pathways, cell growth, and gene expression. However, their overproduction and accumulation, have a negative impact on mitochondria and lead to the formation of more toxic free radical species, such as peroxynitrite and peroxyl radicals, promoting deleterious effects in cells, DNA mutations, disintegration and protein damage of membranes, and alterations in phagocyte-mediated activity, thus playing a pathogenic role in chronic inflammatory diseases. Consequently, inflammation signaling cascades and oxidative stress components are triggered, which in turn enhance the risk of neuropathologies and chronic diseases, such as atherosclerosis, cancer, metabolic syndrome, and, of course, RA [179,180].

Regarding RA, its pathophysiology has been linked to oxidative stress, principally with the Nrf-2 pathway. This is the major pathway involved in the maintenance of homeostatic responses by raising intracellular defense mechanisms and regulating the heme oxygenase-1 axis, controlling macrophage activation and the NF-*κ*B pathway, and reducing stress oxidative and inflammation [181]. Under quiescent conditions, Kelch-like ECH-associated protein 1 (Keap 1) holds Nrf2 in the cytoplasm, promoting its ubiquitination and subsequent proteolysis; however, under pathology conditions, Nrf2 is released from Keap1 and enters the nucleus, where the transcription of antioxidant enzymes occurs [182,183,184].

Among the most promising therapeutic and adjuvant approaches, polyphenols assume a prominent interest since they have already been shown to possess notable capabilities to restore oxidative stress and inflammation near basal levels by interacting with Keap1 protein, avoiding its linkage with its binding site in Nrf-2. This subsequently, causes the dissociation of Keap1 from Nrf2, leading to the transcriptional activation of Nrf-2 and, hence, stimulating intracellular antioxidant enzyme (glutathione, catalase, and superoxide dismutase) activity, and, of course, neutralizing oxidative stress [182,183,184].

**Figure 4:**
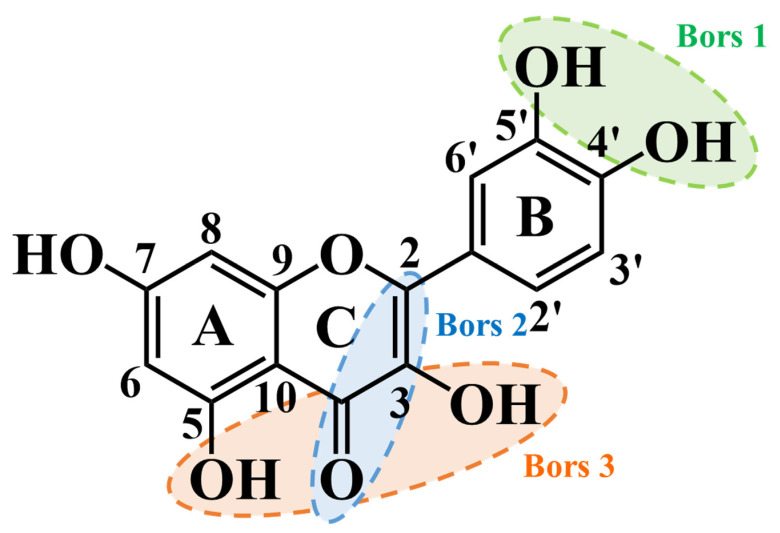
Representation of phenolic antioxidant abilities explained by Bors criteria, involving their structure. Bors 1: catechol group on the B ring (green); Bors 2: 4-oxo group and 2,3 double bond on the C ring (blue); Bors 3: hydroxyl (OH) groups at the 3 and 5 groups on the A and C rings combined with a 4-oxo group on the C ring (adapted from Platzer and co-workers [185] and created with ChemDraw Professional 16.0 (CambridgeSoft, Perkin Elmer Inc., Waltham, MA, USA)).

In general, and in order to establish the antioxidant properties of phenolics, three statements were recently proposed, known as Bors criteria (Figure 4). The first one is the most significant, and it states that because of hydrogen bonding, the presence of a catechol group on the B ring increases the stability of phenolics and, consequently, their antioxidant properties. The second one is the existence of a 2,3 double bond combined with a 4-oxo group on the C ring, which in turn, facilitates electron delocalization and allows for better aryloxyl radical stabilization, while the third one is related to the presence of OH groups at positions 3 and 5 on the A and C rings combined with a 4-oxo group on the C ring, allowing electron delocalization via hydrogen bonds [185]. Thus, these characteristics make phenolics capable of acting as electron and hydrogen donors [55,186]. Among phenolics, quercetin and myricetin complete all Bors criteria, and hence, they are well-known because of their notable capacity to reduce radicals [187]. Moreover, the existence of the catechol group in quercetin and its derivatives enhances their biological potential [185].

Regarding non-flavonoid compounds, hydroxycinnamics showed better antioxidant capacities than hydroxybenzoic acids because of the CH=CH–COOH group, the 7,8-double bond, the carboxyl group with O-alkyl esters, and the presence of hydroxyl groups at the ortho and/or para positions [188,189,190,191]. For lignans, it is well-established that their capacity to eliminate radicals and free species is mainly because of the presence of catechol groups [192], while the hydroxylation at position 4 and hydroxyl groups at the ortho position are the main contributors to the antioxidant potential attributed to stilbenes [193].

Compared with flavonoids, these last ones are more effective in eliminating free species and radicals than non-flavonoids, not only because of the presence of more hydroxyl groups but also because of the double bond in positions 2 and 3, the o-diphenolic group and 3′,4′-catechol hydroxyl groups on the B ring, and the conjugation between the double-bond and the 4-oxo group on the C ring [191,194].

Even so, it is important not to forget that the catechol and pyrogallol groups also make phenolics vulnerable to autoxidation, particularly when they interact with transition metals; however, the glycosylation and methylation of hydroxyl groups can decline this harmful behavior [195,196,197,198]. Furthermore, pH also influences the antioxidant properties of phenolics; in fact, although lower pH levels increase iron-reducing activity, they diminish iron catalytic activity and chelate activities [199].

Focusing on their potential to interact with the Nrf-2 pathway, Diniyah and colleagues [200] revealed that catechin, epicatechin, and gallic acid from non-oilseed legumes present high binding affinities with Keap1, a protein that activates the degradation of Nrf-2, and are therefore promising new Nrf2 activators. Similar results were found involving hesperetin, hesperidin, naringenin, naringin, narirutin, neohesperidin, neohesperidin dihydrochalcone, and nobiletin citrus-derived flavonoids [201] and sesamol isolated from *Sesamum indicum* L. seed oil [183]. Mice treated with 37.5, 75, and 150 mg/kg green tea every 8 h by intragastric administration, which were then sacrificed 4, 12, and 20 h after administration, showed higher levels of antioxidative enzymes and liver phase II enzymes. In addition, docking studies revealed that the most predominant compounds in this tea, which were, caffeine, catechin, catechin gallate, epicatechin, epigallocatechin, epigallocatechin gallate, epicatechin gallate, gallic acid, and gallocatechin, revealed strong binding affinities with the Keap1 protein [184]. In accordance with the mentioned results, the daily intake of 25 mg/kg/body weight (bw) epigallocatechin gallate by rats with lung injury and oxidative stress induced by fluoride displayed the capability to increase antioxidant status and Nrf2 gene expression and reduce inflammatory cytokine [202]. Similar results were obtained using endothelial cells pre-treated with polychlorinated biphenyls 126 [203]. Furthermore, Li et al. [182] verified that concentrations of apigenin, cyanidin 3-sambubioside, echinacoside, luteolin 5-*O*-glucoside, quercetin 3-*O*-rutinoside, and α−tocopherol at 10 and 50 µM can effectively enhance Nrf-2 levels in the nucleus of hydrogen peroxide-induced oxidative-injured rat adrenal pheochromocytoma PC12 cells. It was also demonstrated that 10–100 µM 5-caffeoylquinic acid was a potent Nrf-2 activator in human hepatocellular carcinoma HepG2 cells [204]. Finally, Mishra and colleagues revealed that the combination of curcumin (30 mg/kg/bw) with vitamin E (200 mg/kg/bw) could be very effective in counteracting oxidative stress in hypothyroid rats by interfering with NF-*κ*B/AKT/mTOR/KEAP1 [205].

Moreover, positive correlations were already reported between polyphenol amounts and biological potential. Particularly, positive correlations were already reported between sweet cherry anthocyanin-rich fractions and nitric oxide, superoxide radicals (r > 0.9013) [73], and ferric species (r = 0.739) and between TPCs and non-colored and colored fractions from sweet cherry fruits and hydrogen peroxide (r > 0.940) [79]. Additionally, a high correlation (r = 0.7581) between honey TPC and the elimination of nitric oxide radicals was also found [180,206], and between total flavonoid amounts in sweet cherry vegetal parts and the inhibition of hemoglobin oxidation (r > 0.9636) [207].

Focusing on individual compounds from honey, high correlations were found between caffeoyl hexose, quercetin 7-glucoside-3-*O*-rutinoside, quercetin derivative, and quercetin acetyl rhamnoside and ^●^NO scavenging potential (r > 0.7581), and concerning quercetin 3-*O*-rutinoside and hemoglobin oxidation, hemolysis and lipid peroxidation (r > 0.7355). Additionally, positive correlations were also reported between caffeoyl hexose and quercetin acetyl rhamnoside and lipid peroxidation (r = 0.7352 and r = 0.755, respectively) [206], whereas a mild correlation (r = 0.64) between the quercetin content of pollen extracts and protective effects against oxidative injury induced by AAPH on Hepa1-6 hepatic cells was also reported [208]. On the other hand, strong correlations were found involving quercetin 3-*O*-glucoside (*r* = 0.8640) and *ρ*-coumaric acid derivative 1 (*r* = 0.9444), *ρ*-coumaroylquinic (*r* = 0.8646), *ρ*-coumaric (*r* = 0.8012), and 5-*O*-caffeoylquinic (r = 0.9907) acids in sweet cherries and the nitric oxide scavenging test, and also involving this last hydroxycinnamic and superoxide radical (r = 0.9958) [180,209] as well as between malvidin, delphinidin 3-*O*-arabinoside, and 5-*O*-caffeoylquinic acid and the antioxidant potential shown by blueberry fruits (r > 0.8689) [209].

Considering all the mentioned data, phenolics seem to help prevent and/or attenuate RA by improving oxidative stress-related chronic diseases, chiefly owing to their interaction with the Keap1-Nrf2 complex, thus activating Nrf-2 transcription. Even so, it is important to highlight that, in many cancer types, the activation of Nrf-2 is predominantly associated with drug resistance, which in turn diminishes chemotherapy effects and promotes metastatic invasion of cancer cells [210,211,212]. Thus, several attempts have been made to reduce Nrf-2 levels [213,214]. Indeed, chrysin, luteolin, resveratrol, clofarabine, and 3′,4′,5′,5,7-pentamethoxyflavone have already been shown to possess promising inhibitory activities, revealing half maximal inhibitory concentrations (IC_50_) of 10.20, 1.5–40, 15, 15, and 10–400 µM, as well as the ability to reduce heme-oxygenase 1 [215,216,217,218]. Moreover, agrimoniin isolated from Agrimonia pilosa Aitch (IC_50_ values of 100, 200, 300 μM), galloyl glucoses-1,2,3,4,6-penta-*O*-galloyl-β-D-glucose and 1,3,6-tri-*O*-galloyl-*β*-d-glucose isolated from Excoecaria formosana, schisantherin A isolated from *Fructus schisandrae* (IC_50_ = 2.5 μM), and neferine isolated from the leaves of *Nelumbo nucifera* Gaertn. (IC_50_ = 0–20 μM) and wedelolactone isolated from Eclipta prostrate Lour (IC_50_ = 2.5–20 μM) are also effective in reducing cancer cell proliferation [152,212,214,219,220]. Finally, *Castanea crenata* Siebold and Zucc, *Cinnamomi cortex*, together with procyanidins isolated from it, and rosemary extracts, *Chrysanthemum zawadskii* Herbich and licorice (*Glycyrrhiza uralensis* Fisch. ex DC.), *Bergenia ligulate* (Wall.) Engl. and *Rhododendron luteum* sweet extracts, and strawberry tree honey also showed similar effects [210,211,213,221,222,223,224,225,226], as well as pterostilbene, a natural dimethoxylated analog of resveratrol [227].

### 5.2. Anti-Inflammatory Abilities

During inflammation, IL-1 and IL-6, tumor necrosis factor (TNF-*α*), prostaglandins, heat shock proteins, and nitric oxide and superoxide radicals are generated, which, in part, help in controlling this process by activating neutrophils and macrophages [73]. Hydrogen peroxide is also released, owing to the activation of nuclear factor kappa-light-chain-enhancer of activated B cells (NF-*κ*B) kinases and oxidation, hence stopping reactive species accumulation [228,229]. However, although inflammatory processes are directly linked to individual development, when these responses are exaggerated, carbohydrate damage, lipid peroxidation protein, and nucleic acid oxidation happen, contributing to the onset of many disorders, including AR [230,231,232]. Once again, given that phenolics can counteract oxidative stress and normalize immune responses, it is not surprising that phenolics are a target of extensive research. In fact, they have already been shown to possess capabilities to diminish the activity of COXs, lipoxygenase, and iNOS by interfering with different stages of the inflammatory cascade, namely, by down-regulating NF-*κ*B and activating protein-1 and stimulating Nrf-2, mitogen-activated protein kinase (MAPK), a protein complex responsible for modulating a group of protein kinases that play an essential role in signal transduction by modulating gene transcription in the nucleus, and protein kinase-C [233,234,235,236]. In fact, the inhibition of NF-*κ*B is crucial to attenuate inflammatory responses once this complex of proteins is responsible for controlling the expression of many genes involved in inflammation, including IL-1, IL-2, IL-6, and TNF-*α*, COX-2, vascular endothelial growth factor (VEGF), IL-8, MIP-1α, and MCP-1 chemokines, immuno-receptors, and adhesion molecules. In this way, phenolics can reduce the severity of inflammation [237,238,239].

Within polyphenols, apigenin, catechin, ellagic acid, epigallocatechin gallate, epigallocatechin gallate, homoplantaginin, luteoloside, quercetin aglycone, quercetin 3-*O*-rutinoside, allopurinol, resveratrol, and tangeretin have already shown notable anti-inflammatory effects [203,240,241,242,243,244,245]. Moreover, curcumin, kaempferol 3-*O*-sophoroside, epigallocatechin-gallate, lycopene, and oleanolic acid are effective in inhibiting high mobility group box 1 protein, which is an important chromatin protein involved in the transcription of nucleosomes, transcription factors, and histones related to inflammatory processes [246,247,248,249]. Resveratrol can downregulate the inflammatory pathway activated by TNF-*α* in articular chondrocytes [250].

On the other hand, caffeic acid (10 µg/mL), hydroxybenzoic derivatives (25 µg/mL), *ρ*-coumaric acid (50 µg/mL), and quercetin (100 µg/mL) have already been shown to possess capabilities to suppress MAPK, JNK1 phosphorylation, and NF-*κ*B and hence, downregulate the activity of COX-2 and inducible nitric oxide synthase and, consequently, diminish the production of prostaglandins and nitric oxide radicals, respectively [251,252]. In addition, caffeic acid phenethyl ester can also avoid the activation of toll-like receptor (TLR)-4 activation and liposaccharide-mediated NF-*κ*B in macrophages [253].

Catechin and epicatechin (1.7–17.2 µM) can also modulate phorbol 12-myristate 13-acetate-induced NF-*κ*B activation in Jurkat T cells [254]. Particularly, quercetin can inhibit the biosynthesis of leukotrienes in human polymorphonuclear leukocytes and the activation of NF-*κ*B and p38 MAPK in human mast cells by preventing the degradation of Iκ Bα and the nuclear translocation of p65. Hence, it can reduce IL-1*β*, IL-6, IL-8, and TNF-*α* expression [255] as well as avoid iNOS gene expression by blocking the activation of IkK kinases, NF-*κ*B, and STAT1 in mouse BV-2 microglia [256]. It can also modulate chromatin remodeling by blocking the recruitment of CBP/p300 to the promoters of interferon-inducible protein 10 macrophage inflammatory protein-2 genes in primary murine small intestinal epithelial cells [257]. On the other hand, kaempferol and galangin display identic properties concerning epicatechin effects in mouse BV-2 microglia [258,259].

Similar to epicatechin, epigallocatechin gallate can also avoid IkK kinase activation, avoiding the degradation of Ik-Bα in culture respiratory epithelial cells and in rat models [260] and blocking DNA binding of NF-*κ*B and hence, diminishing IL-12 p40 and iNOS expression in murine peritoneal macrophages and J774.1 macrophage cells [233,261]. Furthermore, 10, 25, 50, and 100 µM epigallocatechin gallate also showed the capacity to attenuate COX-2 expression without affecting COX-1 in colon cancer PC-3 cells [233], as well as the ability to block NF-*κ*B activation in human epithelial cells and reduce iNOS expression and, consequently, nitric oxide radical levels in macrophages at doses of 5 and 10 µM [233]. Furthermore, like genistein [262], luteolin can also suppress the activation of NF-*κ*B and the expression of proinflammatory genes and IKK kinases in murine macrophages RAW 264.7 and mouse alveolar macrophages, as well as TNF-*α* secretion in co-cultured intestinal epithelial Caco-2 and RAW 264.7 cells [263,264].

On the other hand, 25, 50, and 100 µM chebulanim, a natural polyphenol acid isolated from Terminalia chebula Retz inhibited the nuclear translocation of p38 and p65 in liposaccharide-stimulated macrophages in a dose-dependent manner [231]. Additionally, aqueous birch leaf extract of *Betula pendula* Roth inhibits the growth and cell division of inflammatory lymphocytes in a dose-dependent manner because of apoptosis induction [265].

Furthermore, hydroxytyrosol and resveratrol, two polyphenols largely abundant in olive oil and red wine, can also downregulate NF-*κ*B and the expression of vascular cell adhesion molecule-1 in umbilical veins stimulated with liposaccharide at doses varying between 1 and 100 µM/L [266]. Moreover, anthocyanins, namely, cyanidin derivatives (125 µg/mL) demonstrated more efficacy in reducing COX-2 levels than naproxen (10 µM) and ibuprofen (10 µM) (47.4%, 41.3%, and 39.8%, respectively) [267]. In silico tools also revealed high energy bindings of cyanidin 3-*O*-rutinoside (−11.4 kcal/mol), kaempferol 3-*O*-rutinoside (−10.8 kcal/mol), and cyanidin 3-*O*-glucoside (−10.1 kcal/mol) with iNOS [194]. Furthermore, homoplantaginin, luteoloside, quercetin, quercetin 3-*O*-rutinoside, allopurinol, and resveratrol already showed the potential ability, both in vitro and in vivo, to suppress NLRP3 and/or TLR4 inflammasome activation [240,242,243,244,245].

Relative to in vivo studies, the daily ingestion of cherries (141 g) for 10 days by rats and ringdoves downregulated IL-1*β* and TNF-*α* pro-inflammatory cytokine levels and raised IL-4 and IL-2 anti-inflammatory cytokines [268]. In addition, gingerenone A (10 mg/kg/bw), a polyphenol largely present in ginger, also displayed positive effects in suppressing obesity and adipose tissue inflammation by avoiding macrophage infiltration and enhancing adiponectin, in high-fat diet-fed mice that were treated for 15 weeks [269], as well as quercetin [270]. On the other hand, apigenin can stop inflammation in human THP-1-induced macrophages and mouse J 774A, by reducing IL-1*β* production via inhibiting the activation of caspase-1 through the disruption of NLRP3 inflammasome, as well as diminishing TNF-*α* and IL-1*β* thanks to its ability to inactivate NF-*κ*B [241]. Apigenin, as well as other polyphenol compounds extracted from chamomile, meadowsweet, and willow bark, including quercetin and salicylic acid (0–100 μM), also revealed the ability to decrease IL-1*β*, IL-6, and TNF-*α* in THP-1 macrophages and also protect them against oxidative damage [271]. Tannic acid-based nanogel is also an efficient anti-inflammatory agent, showing a notable potential to reduce neutrophil recruitment and pro-inflammatory cytokines, indicating the successful alleviation of inflammation [272].

Moreover, curcumin can easily interfere with NF-*κ*B, as well as with STAT3, thus reducing the expression of TLR-2 and -4 and upregulating peroxisome proliferator-activated receptor ɣ, as observed in male rats that were treated with 0.2 µM curcumin for 3 days [273,274].

Among fruits, sweet cherry phenolic-rich fractions can reduce nitric oxide radicals and decrease iNOS and COX-2 expression in RAW macrophages stimulated with lipopolysaccharide [30]. Moreover, aqueous and hydroethanolic extracts of their vegetal parts also are effective in inhibiting nitrite levels in a dose-dependent manner in these cell lines [28]. Moreover, some Brazilian plants also showed potential to reduce TNF-*α* and CCL2 levels in lipopolysaccharide-stimulated human monocytic THP-1 cells [275]. Regarding individual compounds, ferulic and coumaric acids, which are largely found in Chinese propolis, Lonicera japonica Thunb and *Kalanchoe gracilis* showed similar *capacities* [276,277,278]. In addition, 1–1000 µg/mL quince (Cydonia oblonga Miller) peel polyphenols modulated liposaccharide-induced inflammation in human THP-1-derived macrophages, reducing IL-6 and increasing IL-10 expression by inhibiting NF-*κ*B, p38MAPK and Akt pathways [279].

Focusing on clinical trials, it has already been reported that the daily consumption of cherries (280 g) by women can reduce plasma C-reactive protein and nitric oxide radicals 3 h after intake [280].

#### 5.2.1. Anti-Rheumatoid Effects

As expected, polyphenols and polyphenolic-rich sources also reveal promising abilities to attenuate, or even prevent, rheumatoid arthritis, as described in Table 2.

##### In Vitro Studies

Considering all the previously mentioned results, it is not surprising that polyphenols can be considered promising molecules in preventing and/or attenuating rheumatoid arthritis.

So far, focusing on in vitro assays, it has been already reported that slibinin, a natural flavonoid extracted from milk thistle (*Silybum marianum* L. Gaertner), shows effectiveness in inhibiting Th17 cell differentiation and inducing macrophage M2 polarization in RAW 264.7 cells; it also promotes apoptotic events and inhibits NF-*κ*B, SIRT1, and autophagy in fibroblast-like synoviocytes at doses of 50, 100, and 200 μM [281]. Moreover, 50, 100, and 200 µg/mL oleuropein, the most common polyphenolic detected in olive leaves, showed potential to shift CD4^+^ T cells from peripheral blood mononuclear cells of RA patients to CD4^+^CD25^+^FoxP3 Tregs and induce the production of IL-10 and TGF-β [282]. In addition, 12.5–50 µg/mL polyphenolic extract from extra virgin olive oil inhibits the inflammatory response in IL-1*β*-activated synovial fibroblasts, as well as TNF-*α*, IL-6, COX-2, and microsomal PGE synthase-1 production, thanks to their capability to downregulate the MAPK and NF-*κ*B signaling pathways [283].

Moreover, 5 and 10 µM curcumin showed effectiveness in reducing survivability and decreasing levels of MMP1 and TNF-*α* in synovial sarcoma SW982 cells, which is considered the best in vitro approach to study RA [284]. Additionally, 25–100 µM curcumin was shown to induce apoptosis and inhibit PGE2, by down-regulating anti-apoptotic Bcl-2 and the X-linked inhibitor of the apoptosis protein and upregulating pro-apoptotic Bax expression, in a concentration-dependent manner, on synovial fibroblasts obtained from patients with RA [285]. In addition, at 12.5–50 µM, this compound also showed capacity to reduce IL-1*β*, PMA-induced IL-6, and VEGF-A expression by inhibiting NF-*κ*B and induce dephosphorylation of ERK1/2 and enhance apoptosis in both in MH7A cells and RA-fibroblast-like synoviocytes [286].

On the other hand, 1, 5, and 25 µM quercetin diminish IL-17-stimulated RANKL production in RA fibroblast-like synoviocytes, IL-17-stimulated osteoclast formation, and Th17 differentiation, and hence, modulate bone destructive processes in RA [287]. Furthermore, 12.5–100 µM Punicalagin, a natural polyphenol extracted from pomegranate juice, also showed the capability to reduce IL-1*β*, IL-6, IL-8, IL-17A, MMP-1, and MMP-13 in fibroblast-like synoviocytes [288].

Additionally, 25, 50, 100, 250, and 500 µM syringaldehyde, a small polyphenolic compound extracted from Capparis spinosa L., showed potential to diminish CD86, CD40, MHC II, and IL-23 expression and enhance IL-10 expression and antigen phagocytosis on human acute lymphoblastic leukemia T lymphocytes by inhibiting the MAPK/NF-*κ*B signaling pathways [289]. 

**Table 2:** Main effects attributed to polyphenolic exposition and daily treatment.

Polyphenolic/Plant	Model	Dose	Effects	References
In vitro studies				
Silibinin	RAW 264.7 cells	50, 100, and 200 μM	Th17 cell differentiation inhibition NF-*κ*B, SIRT1, and autophagy inhibition Macrophage M2 polarization induction Apoptotic event promotion	[281]
Oleuropein	Peripheral blood mononuclear cells and of cells of patient with RA	50, 100, and 200 µg/mL	↑ IL-10 and TGF-β Shift CD4^+^ T cells from peripheral blood mononuclear cells of patient with RA to CD4^+^CD25^+^FoxP3 Tregs	[282]
Extra virgin olive oil	Synovial fibroblasts	12.5–50 µg/mL	↓ IL-1*β*, TNF-*α*, IL-6, COX-2, and microsomal PGE synthase-1, and the MAPK and NF-*κ*B signaling pathways	[283]
Curcumin	Synovial sarcoma SW982 cells	5 and 10 µM	↓ MMP1 and TNF-*α*	[284]
Quercetin	RA-fibroblasts-like synoviocytes	1, 5, and 25 µM	↓ IL-17-stimulated RANKL production IL-17-stimulated osteoclast formation Th17 differentiation Modulate bone destructive processes in RA	[287]
Punicalagin	Fibroblast-like synoviocytes	12.5–100 µM	↓ IL-1*β*, IL-6, IL-8, IL-17A, MMP-1, and MMP-13	[288]
Syringaldehyde	Lymphoblastic leukemia T lymphocytes	25, 50, 100, 250, and 500 µM	↓ CD86, CD40, MHC II, and IL-23 ↑ IL-10 and antigen phagocytosis Inhibition of the MAPK/NF-*κ*B signaling pathways	[289]
Resveratrol	Fibroblast-like synoviocytes	1, 3, and 10 µg/mL	↓ Sirt1 protein, MMP1, and MMP13	[290]
Resveratrol	Fibroblast-like synoviocytes	20 µM	Inhibition of phosphorylation and acetylation of p65, c-Jun, and Fos ↓ COX-2 expression	[291]
Resveratrol	Fibroblast-like synoviocytes	1–40 µM	↑ Nrf2-2, heme oxygenase-1, and Bcl-2/Bax, apoptosis ↓ Keap1 expression and ROS and MDA levels Block NF-*κ*B p6 translocation, inhibit cell proliferation and migration	[292,293]
Resveratrol	RSC-364 cells	25 and 50 µmol/L	↓ Hypoxia-inducible factor-1α and activated phosphorylation of p38 MAPK and c-Jun N-terminal kinase Arrest cells at G0/G1 cell-cycle ↑ Apoptosis	[294]
Resveratrol	U251 glioma cells	1–100 µM	Interference with the PI3K/Akt/BAD signaling pathway Inhibition of cells growth and apoptosis	[295,296]
Resveratrol	Human umbilical vein endothelial cells	20 µM	Interference with PI3K/AKT and MEK/ERK Induce FOXO transcriptional activity Inhibition of cell migration and capillary tube formation Prevent angiogenesis	[297]
Resveratrol	Fibroblast-like synoviocytes	50 µM	Block cells at the G2/M stage ↓ TNF-*α* and S phase cell ratio Promote serine–threonine kinase-p53 axis and autophagy Cell apoptosis	[298]
Resveratrol	Human RA synovial MH7A cells	100 and 200 µM	↓ Cell viability Stimulate H2A.X phosphorylation and apoptosis events Mitochondrial membrane potential disruption Stimulate cytochrome c release from the mitochondria to the cytosol Caspase-3 and caspase-9 activation Upregulate the expression of NAD-dependent deacetylase SIRT1 mRNA Downregulate the expression of Bcl-X(L) mRNA Hyperplasia suppression	[299]
Resveratrol	Fibroblast-like synoviocytes	200 µM/L	Caspase-3 activation Inhibition of cell proliferation Induces cell apoptosis	[300]
Resveratrol	Fibroblast-like synoviocytes	25–200 µM	↓ ROS and Bax ↑ Bcl-2 levels and apoptotic cells Regulate the expression of mitochondrial superoxide dismutase	[301]
Resveratrol	Fibroblast-like synoviocytes	100 µM	↓ MMP-1, MMP-3, MMP-9, RANKL, and osteoprotegrin	[302]
Resveratrol	Fibroblast-like synoviocytes	100 µM	↓ TNF-*α* by interfering with the SIRT1/cortistatin pathway	[303]
Resveratrol	Fibroblast-like synoviocytes	100 µM	↑ The expression of genes involved in mitosis, the cell cycle, chromosome segregation, and apoptosis	[304]
Resveratrol	Fibroblast-like synoviocytes	5, 15, and 45 mg/kg	↓ IL-1, IL-6, IL-8, and TNF-*α* ↑ IL-10 and apoptosis	[305]
Resveratrol	Fibroblast-like synoviocytes	10 and 20 µM	↓ Urban particulate matter-induced COX-2/PGE2 release Inhibition of the activation of NADPH oxidase/ROS/NF-*κ*B	[306]
Resveratrol	Mouse preosteoblastic MC3T3-E1 cells	1, 2, 3, and 5 µM	Mediate SIRT-1 interactions with p300 Modulate NF-*κ*B signaling activation Inhibition of osteoclastogenesis Prevent bone loss in bone-derived cells	[307]
Resveratrol + methotrexate	Synovial mononuclear cells from patients with RA	25 µM resveratrol with 0.5 μg/mL methotrexate	↓ Monocyte chemoattractant protein 1 levels	[308]
Curcumin	Fibroblast-like synoviocytes	25–100 µM	Induce apoptosis PGE2 inhibition Downregulate anti-apoptotic Bcl-2 and the X-linked inhibitor of the apoptosis protein Upregulate pro-apoptotic Bax expression	[285]
Curcumin	Fibroblast-like synoviocytes and MH7A cells	12.5–50 µM	↓ IL-1*β*, PMA-induced IL-6 and VEGF-A expression, and cell viability Inhibition of NF-*κ*B and induced dephosphorylation of ERK1/2 ↑ Apoptosis	[286]
Purified grape-derived compounds	1, 10, and 100 µM	Human peripheral blood mononuclear cells	↓ TNF-*α*, IL1, IL-6, and iNOS genes	[150]
Gallotannins	Human mast cells	1, 1, and 10 µg/mL	Downregulate NF-*κ*B expression	[309]
Ellagic acid	Fibroblast-like synoviocytes	10, 25, 50, and 100 µM	↓ IL-6, IL-1*β*, MDA, and TNF-*α* ↑ Superoxide dismutase and apoptosis	[310]
Gallic acid	Fibroblast-like synoviocytes	0.1 and 1 µM	↑ Caspase-3 activity Regulate Bcl-2, Bax, p53, and pAkt production ↓ IL-1*β*, IL-6, CCL-2/MCP-1, CCL-7/MCP-3, COX-2, and MMP-9	[311]
Rosmarinic acid nanoparticles	Macrophages	Not mentioned	↓ RONS and pro-inflammatory cytokines	[237]
*ρ*-Coumaric acid encapsulated with mannosylated liposomes	Macrophages	Not mentioned	↓ RONS and pro-inflammatory cytokines Inhibition of osteoclast differentiation Downregulate the expression of MMP-9 and NFATc1	[312]
Ferulic acid	Fibroblast-like synoviocytes	25–300 µM	↓ IL-17-levels Inhibition of the IL-17/IL-17RA/STAT-3 signaling cascade	[313]
Ferulic acid	RAW 264.7 macrophages	25, 50, and 100 µM	Attenuate RANKL-induced osteoclast differentiation ↓ Bone resorption activity Downregulate NFATc1, c-Fos, TRAP, Cathepsin K, and MMP-9 levels	[314]
Chlorogenic acid	T cells c1	10–50 µg/mL	Inhibit osteoclast differentiation and bone resorption Downregulate RANKL Suppress mRNA expression of NFATc1, TRAP, and OSCAR	[315]
Tea polyphenol carrier-enhanced dexamethasone	Umbilical vein endothelial, murine fibroblast cells L929, and murine macrophage RAW 264.7 cells	Not mentioned	↓ Inflammation	[316]
*Tinospora cordifolia*	RAW 264.7 cells	100, 250, and 500 µg/mL	↓ IL-6, TNF-*α*, PGE2, and NO, and iNOS and COX Modulate JAK/STAT pathway	[317]
Blueberry polyphenols	HIG-82 rabbit synoviocytes	100–200 µM	↓ TNF-*α*, IL-1*β*, MMP3, and NF-*κ*B levels	[318]
Cocoa polyphenols	Mouse epidermal cells	10 and 20 µg/mL	↓ TNF-*α*-induced vascular endothelial growth factor expression Inhibition PI3K and MEK1	[319]
Catechin-7,4′-*O*-digallate from *Woodfordia uniflora*	Mouse macrophages	5–80 µM	↓ IL-6 and IL-1*β* levels Regulate the NF-*κ*B signaling pathway	[320]
*Salacia reticulata* leaves	MTS-C H7 cells	IC_50_ score of ~850 μg/mL	Inhibition of cell proliferation	[321]
In vivo studies				
Silibinin	Rats with induced RA	50, 100, and 150 mg/kg	↓ IL-1*β*, IL-6, and TNF-*α* levels and joint inflammation	[281]
Resveratrol	Rats with induced RA	5 mg/kg, 15 mg/kg, and 45 mg/kg	↓ Abnormal proliferation of fibroblast-like synoviocytes, swelling degree of the paw, and malondialdehyde levels ↑ Superoxide dismutase activity, glutathione peroxidase, and the glutathione reductase ratio	[322]
Resveratrol	Rats with induced RA	10 mg/kg	↓ Progression of periodontitis and rheumatoid factor amount	[228]
Resveratrol	Rats with induced RA	10 mg/kg	↓ Wnt5a, MAPK3, Src kinase, and STAT3 levels	[323]
Resveratrol	Rats with induced RA	10 mg/kg	↓ IL-6 and TNF-*α* levels, atrial apoptosis and fibrosis, and activate the AMPK/PGC-1α pathway	[324]
Resveratrol	Rats with induced RA	10 mg/kg	↓ Serum rheumatoid factor, MMP-3, cartilage oligomeric matrix protein, IgG, antinuclear antibody, TNF-*α*, MPO, C-reactive protein, and MDA ↑ IL-10 and glutathione	[325]
Resveratrol	Rats with induced RA	50 mg/kg	↓ Paw swelling, TNF-*α*, IL-1*β*, TBARs, and NOx Suppress NF-*κ*B p65 expression	[326]
Resveratrol	Rabbit inflammatory RA model	10 µMol/kg	↓ Inflammatory responses Prevent the loss of matrix proteoglycan content in the cartilage	[327]
Resveratrol	Murine collagen-induced arthritis	15 and 20 mg/kg	Inhibition of Th17 and B-cell function	[328]
Resveratrol	Rats with bovine type-II collagen-induced arthritis	400 g/kg/bw	↓ Oxidative stress, inflammation, and MDA levels ↑ Serum superoxide dismutase Suppress MAPK signaling pathways and angiogenesis	[294]
Resveratrol	Adjuvant arthritis rat model	45 mg/kg	↓ Store-operated Ca^2+^ entry ↑ Apoptosis Interference with the ORAI1-STIM1 complex	[329]
Resveratrol	Rats with induced RA	12.5 mg/kg	Induce the noncanonical autophagy pathway ↓ p62 expression, caspase-3 expression, poly(ADP-ribose) polymerase, IL-1*β*, C-reactive protein, prostaglandin E2, and NF-*κ*B synovial tissue expression	[330]
Resveratrol	Rats with induced RA	12.5 mg/kg	↓ PCNA, CD68, CD3, monocyte chemoattractant protein-1 staining, cytokine-induced neutrophil chemoattractant-1, and the level of the marker of DNA damage, 8-oxo-7,8-dihydro-2′-deoxyguanine	[331]
Resveratrol	Collagen-induced arthritis rat model	2.5 and 10 mg/kg	Suppress MMP1 and MMP13 amounts	[290]
Resveratrol	Adjuvant arthritis rats	10 and 50 mg/kg	↓ The proliferation of concanavalin A-stimulated spleen cells, articular cartilage degeneration with synovial hyperplasia and inflammatory cell infiltration Suppress the production of COX-2 and PGE2	[332]
Resveratrol	Rats with induced RA	10 mg/kg	Alleviates adjuvant arthritis-interstitial lung disease	[333]
Resveratrol	Rats with induced RA	10 mg/kg	Prevent the production of pro-inflammatory by modulating JAK/STAT/RANKL signaling pathway Ameliorate fibrosis via the autophagy lysosome pathway	[232]
Resveratrol combined with methotrexate loaded-nanoemulsion	Rats with induced RA	Not mentioned	↓ Inflammation Better anti-arthritic effects potentiated by resveratrol	[334]
QRu-PLGA-DS nanoparticles carried resveratrol	Arthritic rats	Not mentioned	Improves the water solubility and targets the effectiveness of this compound Ameliorate anti-inflammatory effects ↑ M2-type macrophage transformation ↓ The recruitment of M1-type macrophages	[335]
Ellagic acid	Arthritic rats	5, 50, and 100 mg/kg	↓ Oxidative stress and inflammation ↑ Serum superoxide dismutase Suppresses MAPK signaling pathways, angiogenesis, and MTA1/HDAC1-mediated Nur77 deacetylation	[310]
Ellagic acid	Arthritic rats	25 mg/kg	↓ Articular edema, NF-*κ*B, neutrophil elastase, and neutrophil extracellular traps Interference with TLR-4, peptidyl arginine deiminase 4 enzyme, and COX-2	[336]
Epigallocatechin gallate	Rats with induced RA	10 mg/kg	Ameliorate RA symptoms ↓ Histological scores in arthritic mice, as well as reduces IgG2a antibodies Suppress T cell proliferation and relative frequencies of CD4 T cells, CD8 T cells, and B cell subsets ↑ The frequency of CD4^+^-Foxp3^+^ Treg cells and indoleamine-2,3-dioxygenase expression by CD11b^+^ dendritic cells, NF-*κ*B, Nrf-2, and heme oxygenase-1	[337]
Epigallocatechin gallate	Collagen-induced arthritis rat model	50 mg/kg	↓ TNF-*α*, IL-17, Nrf-2, and MDA levels ↑ Heme oxygenase-1, superoxide dismutase, catalase, and glutathione peroxidase levels	[234]
Epigallocatechin gallate	Rats with induced RA	10 mg/kg	↓ Neuroinflammation, namely, by activating caspase-3	[338]
Epigallocatechin gallate	Mice with collagen-induced arthritis	50 mg/kg	↓ The arthritis index Protective effects against joint destruction Inhibition of osteoclastogenesis and TH17 cell activation ↑ The number of Treg cells	[339]
Extracellular vesicles-encapsulated epigallocatechin gallate	Rats with induced RA	Not mentioned	Downregulate the expression of hypoxia-inducible factor 1-α Inhibition apoptosis of chondrocytes Promote the recovery of type II collagen ↓ Joint swelling	[340]
Epigallocatechin	Arthritic rats	Not mentioned	↑ Reduced elastic modulus, hardness, and stiffness in cartilage	[341]
Epigallocatechin	Rats with induced RA	10 mg/kg	Prevent cartilage destruction in at by imbibing myeloperoxidase activity	[342]
Green tea	Rats with induced RA t	2–12 g/L	↓ RA severity and IL-17 levels ↑ IL-10 levels Suppress the anti-Bhsp65 antibody response	[343]
*Tinospora cordifolia*	Rats with induced RA	150 mg/kg	↓ Erythema, paw edema, hyperplasia, IL-6, TNF-*α*, IL-17, NO, and PGE2 levels, phosphorylation of STAT3, and the expression of VEGF	[317]
Kalpaamruthaa	Rats with induced RA	150 mg/kg	↓ Oxidative stress, myeloperoxidase and lipid peroxide, and increase the activity of enzymic and non-enzymic antioxidants	[344]
*Ribes orientale* Def.	Sprague Dawley rats with induced RA	50, 100, and 200 mg/kg	↓ Paw volume/diameter and PGE2, COX-2, IL-1*β*, IL-6, NF- kB, and TNF-*α* levels ↑ IL-4 and IL-10	[229]
Chebulanin	Collagen-induced arthritis mouse model	80 mg/kg	Suppress the progression and development of RA ↓ Arthritis severity scores, paw swelling and joint destruction, IL-6 and TNF-*α* amounts and excised phosphorylated (p)-p38 and p-p65, phosphorylated-c-JUN N-terminal kinase, and phosphorylated NF-*κ*B and inhibitor alpha	[231]
Punicalagin	Rats with induced RA	50 mg/kg/	Prevent the translocation of p-65 Avoid the phosphorylation of IkK and Ik B*α* Modulate the NF-*κ*B pathway ↓ TNF*α*, IL-6, CD86, CCR7, CD40, and MHC II expression, Th1, Th17, and Th17/Th1-like ↑ IL-10 expression Suppress dendritic cell migration Promote the generation of Tregs via the regulation of dendritic cells maturation	[288]
Syringaldehyde	Rats with induced RA	10, 25, and 50 mg/kg	Alleviate paw and joint edema ↓ TNF-*α* and IL-6 levels ↑ IL-10	[289]
Syringaldehyde	Rats with induced RA	100 and 200 mg/kg	↓ IL-6 and TNF-*α* levels	[345]
*Clitoria ternatea* L. flower petals and its major compound, quercetin-3ß-D-glucoside	Rats with induced RA	50 mg/kg *Clitoria ternatea* L. flower petals and 2.5 mg/kg of quercetin-3ß-D-glucoside	↓ MPO activity and pro-inflammatory cytokines, chemokines, RNOS, and TNFR1, TLR2, iNOS, COX-2, and MMP-2 expression levels	[346]
*Berberis orthobotrys* Bien ex Aitch	Rats with induced RA	150 mg/kg	Protected against arthritic lesions, oxidative damage, and body weight alterations Ameliorated altered hematological parameters and the rheumatoid factor Contributed to positively modified radiographic and histopathological changes	[347]
*Diospyros malabarica* (Desr.) Kostel fruits	Rats with induced RA	250, 500, and 750 mg/kg	↑ Anti-inflammatory enzymes ↓ Anti-inflammatory enzymes	[348]
*ρ*-Coumaric acid	Rats with induced RA	100 mg/kg	Suppress paw edema and body weight loss ↓ cartel	[316]
*ρ*-Coumaric acid	Rats with induced RA	100 mg/kg	↓ Age, bone erosion, TNF-*α*, IL-1*β*, IL-6, IL-17, and MCP-1, and the expression of RANKL and TRAP, *i*NOS and COX-2, JNK, p-JNK, and ERK1/2 Regulate RANKL/OPG imbalance Inhibit RANKL-induced NFATc-1 and c-Fos expression	[238,349]
Chlorogenic acid	Rats with induced RA	10 mg/kg	Attenuate liposaccharide-induced bone loss of rat femurs	[315]
Theaflavin-3,3′-digallate	Collagen-induced RA mouse model	10 mg/kg	↓ IL-1*β*, TNF-*α*, IL-6, as well as MMP-1, MMP-2, and MMP-3 amounts Inhibition the activation of NF-*κ*B and the phosphorylation of P38, JNK2, and ERK	[230]
Cinnamtannin D1	Rats with induced RA	50 mg/kg	Alleviate the severity of RA ↓ Clinical scores and paw swelling, inflammatory cell infiltration, cartilage damage in joints, IL-17, IL-6, and IL-1*β* levels, and the frequency of Th17 cells ↑ TGF-*β* and IL-10 levels and the frequence of Treg cells Inhibition of aryl hydrocarbon receptor expression and phospho-STAT3/RORγt	[350]
Cinnamon barks	Mice with induced RA	200 mg/kg	↓ Paw volume, weight loss, and IL-2, IL-4, and IFNγ levels	[351]
N-feruloylserotonin	Rats with induced RA	3 mg/kg	↓ C-reactive protein, the activity of LOX, as well as mRNA transcription of TNF-*α*, iNOS IL-1*β*, and IL-1*β* mRNA expression	[352]
Extra virgin olive oil	Mice with collagen-induced RA	100 and 200 mg/kg	↓ Inflammatory markers, joint edema, cell migration, cartilage degradation and bone erosion, and also reduces COX-2 and microsomal prostaglandin E synthase-1 expression Inhibition c-Jun N-terminal kinase, p38, signal transducer, and activator of transcription-3	[353]
Hydroxytyrosol acetate	Mice with collagen-induced RA	0.05%	↓ IgG1 and IgG2a, COMP, MMP-3, TNF-*α*, IFN-*γ*, IL-1*β*, IL-6 and IL-17A, and MAPKs JAK/STAT and NF-*κ*B pathways ↑ Nrf-2 and heme oxygenase-1	[354]
Mangiferin	Mice with induced RA	50, 100, and 400 mg/kg	Inhibition of mRNA expression of cytokine genes in the thymus and spleen, and also NF-*κ*B and activating ERK1/2 ↓ IL-1*β*, IL-6, TNF-*α*, and RANKL	[355]
*Sarcococca saligna*	Rats with induced RA	250 mg/kg	↓ IL-1*β*, IL-6, COX-2, prostaglandin E2, TNF-*α*, and NF-*κ*B levels, the arthritic index, and paw inflammation ↑ IL-4 and IL-10 levels	[236]
Curcumin	Rats with induced RA	10 mg/kg	↓ TNF-*α* and IL-1*β*	[356]
*Dichrostachys cinerea* Wight et Arn. fruits	Rats with induced RA	75.48 mg	↓ IL-1*β*, IL-6, TNF-*α*, and cortisol levels, lipid peroxidation, and NOx production	[357]
*Circaea mollis* Sieb. and Zucc. plant	Freund’s complete adjuvant-induced arthritis model in rats	170–1350 mg/kg	↓ Paw and inflammatory swelling, the arthritis index, TNF-*α*, and IL-1*β* levels ↑ IL-10 levels	[358]
*Opuntia littoralis*	Rats with induced RA	10 and 20 mg/100 g bw	↓ Joint inflammation, paw swelling, edemas, MDA, and IL-1*β*, IL-6R, IL-6, IL-17, and IL-23, Ameliorate COX-2, NF-*κ*B, STAT-3, PTEN, and RANKL expression Upregulate the expression of miR-28 and miR-199a	[239]
*Antrocaryon micraster* A. Chev. and Guillaumin seeds	Rats with induced RA	25 and 100 mg/kg	↓ Cachexia, paw edema, infiltration of inflammatory cells, pannus formation, and synovium damage	[359]
Dried plums	Transgenic mice with induced RA	+ 20% dried plums in the normal diet	Protect articular cartilage ↓ Synovitis, IL-1*β*, MCP1, MIP1*α*, MMP1 and MPP3, and RANKL expression Repress TNF-induced formation of osteoclasts and mRNA levels of cathepsin K and MMP9 Inhibition of NFATc1 expression and NF-*κ*B activation	[360]
*Opuntia monacantha*	Rats with induced RA	750 mg/kg	↓ Paw edema, the arthritic score, the rheumatoid factor, inflammation, COX-2, IL-6, TNF-*α*, IL-1, NF-*κ*B, bone erosion, and pannus formation Restore hemoglobin, white blood count, and platelet parameters ↑ Catalase and superoxide dismutase, IL-4 and IL-10 levels Inhibition of glutaminase 1 activity	[361]
*Solanum nigrum*	Rats with induced RA	800 mg/kg	↓ Paw edema Restore body weight, hematologic parameters, and radiographic and histopathologic alterations	[362]
Quercetin and quercetin-loaded chitosan	Rats with induced RA	15 mg/kg quercetin and 10 and 20 mg/kg quercetin-loaded chitosan	↓ TNF-*α* and IL-6 The nanoencapsulation of quercetin enhances its efficacy	[363]
Grape polyphenols + propolis	Female rats with induced RA	1.25 g/kg grape polyphenols mixed with 1.25 g/kg propolis	↓ The intensity of cachexia and alleviate RA scores	[364]
Malvidin 3-*O*-*β* glucoside	Chronic rat adjuvant-induced arthritis with	125 mg/kg	↓ Cachexia and arthritic paw scores	[150]
*Phoenix dactylifera* L. seeds	Rats with induced RA	30 mg/kg	↓ IL-1*β* levels, paw edema, the erythrocyte sedimentation rate, and C-reactive protein	[365]
Liposomal drug delivery system for morin	Rats with induced RA	Not mentioned	↓ TNF-*α*, IL-1*β*, IL-6, IL-17, RANKL, STAT-3, p-STAT-3, VEGF, iNOS, and NF-*κ*B-p65 ↑ Osteoprotegerin and murin uptake by rats synovial and spleen macrophages	[235]
Clinical trials				
Low-calorie cranberry juice	500 mL/day	Women with RA	↓ Anti-cyclic citrullinated peptide antibodies levels, pain intensity, and swollen joints	[366]
Low-calorie cranberry juice + fish oil *ω*-3 fatty acids	500 mL/day of low-calorie cranberry juice with 3 g of fish oil *ω*-3 fatty acids	People with rheumatoid arthritis	↓ C-reactive protein, the erythrocyte sedimentation rate, and related pain	[367]
Pomegranate extract	250 mg	Patients with RA	↓ Swollen, pain intensity and tender joints, the erythrocyte sedimentation rate, and morning stiffness ↑ Glutathione peroxidase	[368]
Resveratrol	1 g	Patients with	↓ Joint swelling, tenderness, TNF-*α*, IL-6, protein C-reactive, MMP-3, the erythrocyte sedimentation rate, and undercarboxylated osteocalcin	[369]

↑: enhance; ↓ diminish; IL: interleukin; ROS: reactive oxygen species; RONS: reactive oxygen and nitrogen species; NO: nitric oxide; TNF: tumor necrosis factor; MMP: matrix metalloproteinase; SIRT: sirtuin; MDA: malondialdehyde; iNOS: inducible nitric oxide synthase; VEGF: vascular endothelial growth factor; STAT: signal transducers and activators of transcription; RANKL: receptor activator of nuclear factor kappa beta; RA: rheumatoid arthritis; NF-*κ*B: nuclear factor kappa B; MPO: myeloperoxidase; COX: cyclooxygenase; JNK: c-Jun N-terminal kinase; BAX: Bcl-2 associated X protein; Bcl-2: B-cell lymphoma 2; MAPK: mitogen-activated protein kinase; ERK: extracellular signal-regulated kinase.

Furthermore, 1, 3, and 10 µg/mL resveratrol also showed ability to reduce Sirt1 protein, MMP1 and MMP13 expression [290]. In addition at 20 µM, it inhibited the phosphorylation and acetylation of p65, c-Jun, and Fos and reduced binding to the COX-2 promoter, thereby attenuating COX-2 expression in fibroblast-like synoviocytes [291]. In addition, at 1–40 µM, this compound also showed an ability to activate Nrf2-2, heme oxygenase-1, and Bcl-2/Bax; induce apoptosis; reduce Keap1 expression and reactive oxygen species and malondialdehyde levels; block NF-*κ*B p6 translocation; inhibit cell proliferation; and migration on fibroblast-like synoviocytes in a dose-dependent manner [292,293]. Moreover, Yang and co-workers [294] demonstrated that 25 and 50 µmol/L resveratrol can reduce hypoxia-inducible factor-1α and activate phosphorylation of p38 MAPK and c-Jun N-terminal kinase in IL-1*β*-stimulated RSC-364 cells, as well as arrest these cells at the G0/G1 cell cycle and enhance their apoptosis. Similar data were reported by Tian et al. [295,296], who also verified that resveratrol can interfere with the PI3K/Akt/BAD signaling pathway, which, consequently, also promotes the inhibition of cells growth and apoptosis. In addition to that, 20 µM resveratrol can also interfere not only with PI3K/AKT but also with the MEK/ERK pathway, and thus induce FOXO transcriptional activity and inhibit cell migration and capillary tube formation in human umbilical vein endothelial cells, preventing angiogenesis [297]. On the other hand, Li et al. [298] reported that 50 μM resveratrol can block fibroblast-like synoviocytes in RA cells at the G2/M stage and reduce TNF-*α* levels and the S phase cell ratio by promoting the serine–threonine kinase-p53 axis, and autophagy, which, subsequently, leads to cells apoptosis. Moreover, 100 and 200 µM resveratrol also showed the ability to reduce the viability of human RA synovial MH7A cells by stimulating H2A.X phosphorylation and consequent apoptosis events, disrupt mitochondrial membrane potentials and stimulat cytochrome c release from the mitochondria to the cytosol, activate caspase-3 and caspase-9 but not caspase-8, upregulate the expression of the NAD-dependent deacetylase sirtuin (SIRT) 1 mRNA and downregulate the expression of the Bcl-X(L) mRNA, hence suppressing synovial cells hyperplasia [299]. The capacity of resveratrol to activate caspase-3, and consequently inhibit the proliferation of synoviocytes and induce cell apoptosis in synoviocytes in RA, was already reported by Tiang et al. [300]. Furthermore, Wang and colleagues revealed that 25–200 µM of this compound can also reduce mitochondrial reactive oxygen species and Bcl-2-associated X protein (Bax) and increase B-cell-lymphoma-2 (Bcl-2 levels) and apoptotic cells, namely, by regulating the expression of mitochondrial superoxide dismutase [301]. On the other hand, a dose of 100 µM resveratrol reduces the expression of MMP-1, MMP-3, MMP-9, RANKL, osteoprotegrin [302], and TNF-*α* by interfering with the sirtuin1/cortistatin pathway [303], as well as increasing the expression of genes involved in mitosis, the cell cycle, chromosome segregation, and apoptosis in RA fibroblast-like synoviocytes [304]. Similar data were obtained by Lu and co-workers [305], who also verified that resveratrol can diminish the expression of IL-1, IL-6, IL-8, and TNF-*α* and raise the expression of IL-10; in addition, the administration of resveratrol together with 5 µM hydrogen peroxide can induce fibroblast-like synoviocyte apoptosis probably via mitochondrial dysfunction and endoplasmic reticulum stress. Resveratrol can also diminish urban particulate matter-induced COX-2/PGE2 release in human fibroblast-like synoviocytes by inhibiting the activation of NADPH oxidase/ROS/NF-*κ*B [306]. Finally, 1, 2, 3, and 5 µM resveratrol can mediated SIRT-1 interactions with p300, modulating RANKL activation of NF-*κ*B signaling, inhibiting osteoclastogenesis, and thus, preventing bone loss in bone-derived cells [307].

In addition, it was also already reported that the combination of 25 µM resveratrol with 0.5 μg/mL methotrexate significantly reduced monocyte chemoattractant protein 1 levels in synovial mononuclear cells from patients with RA [308].

It was previously shown that 1, 10, and 100 µM purified grape-derived compounds, whose main compound is malvidin 3-*O*-*β* glucoside, showed capacity to inhibit the expression of TNF-*α*, IL1, IL-6, and iNOS genes from secretion-activated macrophages of human peripheral blood mononuclear cells [150].

Furthermore, 1, 1, and 10 µg/mL 1,2,3,4,6-penta-*O*-galloyl-beta-D-glucose, 1,2,6-tri-*O*-galloyl-beta-D-allopyanose, and 1,2,3,6-tetra-*O*-galloyl-beta-D-allopyranose gallotannins also showed the potential to downregulate NF-*κ*B expression in a dose dependent in human mast cells [309].

Focusing on phenolic acids, 10, 25, 50, and 100 µM ellagic acid showed potential to reduce IL-6, IL-1*β*, malondialdehyde, and TNF-*α* and raise superoxide dismutase and apoptosis in fibroblast-like synoviocyte MH7A cells pre-treated with TNF-*α* to induce inflammation by inhibiting metastasis-associated gene 1, which is a component of NF-*κ*B signaling and a upstream modulator of inflammation and immunologic responses [310]. On the other hand, 0.1 and 1 µM gallic acid increase caspase-3 activity, regulate Bcl-2, Bax, p53, and pAkt production and reduce IL-1*β*, IL-6, CCL-2/MCP-1, CCL-7/MCP-3, COX-2, and MMP-9 on fibroblast-like synoviocytes from patients with RA [311]. More recently, it was reported that rosmarinic acid nanoparticles showed a favorable capability in scavenging reactive oxygen and nitrogen species and pro-inflammatory cytokines produced by macrophages [237]. *ρ*-Coumaric acid encapsulated with mannosylated liposomes showed similar effects, as well as an ability to inhibit osteoclast differentiation and downregulate the expression of MMP-9 and NFATc1 [312]. Moreover, 25–300 µM ferulic acid showed ability to reduce IL-17-levels in RA fibroblast-like synoviocytes thanks to its ability to inhibit the IL-17/IL-17RA/STAT-3 signaling cascade [313]. In addition, this acid at doses of 25, 50, and 100 µM can attenuate RANKL-induced osteoclast differentiation and, consequently, decrease bone resorption activity by downregulating NFATc1, c-Fos, TRAP, Cathepsin K, and MMP-9 levels in RAW 264.7 macrophages [314]. On the other hand, 10–50 µg/mL chlorogenic acid can inhibit osteoclast differentiation and bone resorption by downregulating RANKL on activated T cells c1, as well as by suppressing mRNA expression of NFATc1, TRAP, and OSCAR [315].

Tea polyphenol carrier-enhanced dexamethasone showed an ability to reduce inflammatory mediators in LPS/INF-γ-induced inflammatory cell models, including umbilical vein endothelial, murine fibroblast cells L929, and murine macrophage RAW 264.7 cells [316]. *Tinospora cordifolia* (Willd.) Hook. f. and Thomson also showed anti-inflammatory effects on lypossacharide-stimulated RAW 264.7 cells by reducing IL-6, TNF-*α*, PGE2, and nitric oxide levels and iNOS and COX expression via modulation of the JAK/STAT pathway [317]. On the other hand, 100–200 µM blueberry polyphenols can reduce TNF-*α*, IL-1*β*, MMP3, and NF-*κ*B levels on HIG-82 rabbit synoviocytes previous stimulated with TNF-*α* [318]. Moreover, 10 and 20 µg/mL cocoa polyphenols can suppress TNF-*α*-induced vascular endothelial growth factor expression by inhibiting phosphoinositide 3-kinase (PI3K) and mitogen-activated protein kinase kinase-1 (MEK1) activities in mouse epidermal cells [319]. Another study showed that 5–80 µM Catechin-7,4′-*O*-digallate from *Woodfordia uniflora* can also regulate the NF-*κ*B signaling pathway in mouse macrophages and reduce IL-6 and IL-1*β* levels [320]. On the other hand, Salacia reticulata leaves showed the capacity to inhibit MTS-CH7 cell proliferation (IC_50_ score of ~850 µg/mL) [321].

##### In Vivo Studies

On the other hand, in RA-mice models, 50, 100, and 150 mg/kg/day silibinin showed an ability to diminish IL-1*β*, IL-6, and TNF-*α* levels and joint inflammation [281].

Resveratrol was shown to be a promising strategy for attenuating RA in a mice model. In fact, a 12-day treatment with 5 mg/kg, 15 mg/kg, and 45 mg/kg resveratrol can reduce the abnormal proliferation of fibroblast-like synoviocytes, swelling degree of the paw, and malondialdehyde levels and increase superoxide dismutase activity, glutathione peroxidase, and the glutathione reductase ratio [322]. Furthermore, at 10 mg/kg, this compound showed an ability to reduce the progression of periodontitis and the rheumatoid factor amount [228], Wnt5a, MAPK3, Src kinase, and STAT3 levels [323], diminish IL-6 and TNF-*α* levels and atrial apoptosis and fibrosis, and activate the AMPK/PGC-1α pathway [324], as well as decrease the serum rheumatoid factor, MMP-3, cartilage oligomeric matrix protein, IgG, antinuclear antibody, TNF-*α*, myeloperoxidase, C-reactive protein and malondialdehyde to about 37, 59, 44, 70, 5, 30, 23, 33, and 28%, respectively, and enhance IL-10 and glutathione to about 225 and 273%, respectively, in RA-rats [325]. Furthermore, 50 mg/kg resveratrol can diminish paw swelling, TNF-*α*, IL-1*β*, TBARs, and NOx, namely, by suppressing NF-*κ*B p65 expression [326], while 10 µMol/kg can reduce inflammatory responses and prevent the loss of matrix proteoglycan content in the cartilage in a rabbit inflammatory arthritis model [327]. Moreover, a 10-day treatment with 15 and 20 mg/kg resveratrol modulates murine collagen-induced arthritis by inhibiting Th17 and B-cell function [328]. In accordance with other studies, Yang et al. [294] also showed that 400 g/kg/bw resveratrol diminishes oxidative stress and inflammation, by raising serum superoxide dismutase and reducing malonaldehyde, and suppresses the MAPK signaling pathways and angiogenesis in rats with bovine type-II collagen-induced arthritis. Similar results were observed in arthritic rats treated with 5, 50, and 100 mg/kg ellagic acid for 21 days, where ellagic acid also showed a capacity to suppress MTA1/HDAC1-mediated Nur77 deacetylation [310]. Furthermore, 25 mg/kg of this compound can also decrease articular edema, NF-*κ*B, and neutrophil elastase, and hence, diminish neutrophil extracellular traps, in RA rats, namely, by interfering with TLR-4, peptidyl arginine deiminase 4 enzyme and COX-2 [336]. Resveratrol at 45 mg/kg can also reduce store-operated Ca^2+^ entry and enhance the apoptosis in an adjuvant arthritis rat model by targeting the ORAI1-STIM1 complex [329]. Additionally, Fernández-Rodríguez et al. [330] reported that 12.5 mg/kg/day resveratrol can also induce the noncanonical autophagy pathway, reduce p62 expression, caspase-3 expression, and poly(ADP-ribose) polymerase, IL-1*β*, C-reactive protein, and prostaglandin E2, as well as NF-*κ*B synovial tissue expression, which is correlated with p62 expression [330]. It can also diminish PCNA, CD68, CD3, monocyte chemoattractant protein-1 staining, cytokine-induced neutrophil chemoattractant-1 and the level of the marker of DNA damage, and 8-oxo-7,8-dihydro-2′-deoxyguanine in RA rats [331]. Resveratrol at 2.5 and 10 mg/kg/day can also display an ability to suppress MMP1 and MMP13 amounts in a collagen-induced arthritis rat model [290], while 10 and 50 mg/kg resveratrol can reduce the proliferation of concanavalin A-stimulated spleen cells, articular cartilage degeneration with synovial hyperplasia, and inflammatory cell infiltration, namely, by suppressing the production of COX-2 and PGE2 in adjuvant arthritis rats [332]. Resveratrol at 10 mg/kg/day alleviates adjuvant arthritis-interstitial lung disease in rats with induced RA, namely, by preventing the production of pro-inflammatory by modulating the JAK/STAT/RANKL signaling pathway [333], as well as ameliorate fibrosis in rats with induced-RA via the autophagy-lysosome pathway [232].

More recently, to improve resveratrol’s bioavailability and effectiveness, it was shown that its combination with methotrexate loaded-nanoemulsion in a transdermal delivery system resulted in a reduction in inflammation by 78.76% and a better anti-arthritic effects [334]. Moreover, the nanocarrier QRu-PLGA-DS nanoparticles effectively improved the water solubility and targeting ability of this compound to reverse M1 to M2 type macrophages and ameliorate its anti-inflammatory effects in arthritic rats. In addition, the accumulation of nanoparticles in the lesion area with an exogenous stimulus significantly raises the transformation of M2 type macrophages and decreases the recruitment of M1 type macrophages [335].

Regarding epigallocatechin gallate, 10 mg/kg can ameliorate clinical symptoms and reduce histological scores in arthritic mice, as well as reduce IgG2a antibodies, suppress T cell proliferation and relative frequencies of CD4 T cells, CD8 T cells, and B cell subsets, including marginal zone B cells, T1 and T2 transitional B cells, and increase the frequency of CD4^+^-Foxp3^+^ Treg cells and indoleamine-2,3-dioxygenase expression by CD11b^+^ dendritic cells, NF-*κ*B, Nrf-2, and heme oxygenase-1 [337]. On the other hand, in a collagen-induced arthritis rat model, 50 mg/kg/day epigallocatechin gallate also showed the capacity to diminish TNF-*α*, IL-17, Nrf-2, and malondialdehyde levels and improve heme oxygenase-1, superoxide dismutase, catalase, and glutathione peroxidase levels [234]. Moreover, it can also reduce neuroinflammation in RA rats after 2 months of treatment, namely, by activating caspase-3 [338]. Moreover, 50 mg/kg epigallocatechin gallate showed the ability to decrease the arthritis index, provide protective effects against joint destruction in mice with collagen-induced arthritis, inhibit osteoclastogenesis and TH17 cell activation, and increase the number of Treg cells [339]. On the other hand, the administration of extracellular vesicle-encapsulated epigallocatechin gallate for cartilage repair in rat arthritis downregulated the expression of hypoxia-inducible factor 1-α, inhibited apoptosis of chondrocytes, promoted the recovery of type II collagen, and reduced joint swelling in rheumatoid rats by approximately 39.5% [340]. In addition, the nanoindentation of epigallocatechin significantly increased reduced elastic modulus (57.5%), hardness (83.2%), and stiffness (17.6%) in the cartilage of arthritic rats [341]. It also showed the capability of preventing cartilage destruction in rats with induced-RA at 10 mg/kg by imbibing myeloperoxidase activity [342].

Regarding green tea, it was also reported that rats with induced RA that ingested 2–12 g/L in drinking water for 1–3 weeks showed significant reductions in RA severity, lower levels of IL-17, and higher levels of IL-10. This is probably due to the capacity of green tea polyphenols to suppress the anti-Bhsp65 antibody response [343].

Another study showed that 150 mg/kg *Tinospora cordifolia* (Willd.) Hook. f. and Thomson reduced erythema, paw edema, hyperplasia, IL-6, TNF-*α*, IL-17, nitric oxide, and PGE2 levels, as well as phosphorylation of STAT3 and the expression of VEGF [317].

Furthermore, 150 mg/kg/bw Kalpaamruthaa, a modified indigenous Siddha preparation constituting *Semecarpus anacardium* nut milk extract, *Emblica officinalis*, and honey, also showed the potential to reduce oxidative stress, myeloperoxidase, and lipid peroxide and increase the activity of enzymic and non-enzymic antioxidants in arthritic rats [344].

In addition, 50, 100, and 200 mg/kg doses of *Ribes orientale* Desf. aqueous ethanolic extract (30:70) showed potential to reduce paw volume/diameter and PGE2, COX-2, IL-1*β*, IL-6, NF-*κ*B, and TNF-*α* levels and enhance IL-4 and IL-10 contents in Sprague Dawley rats with induced RA [229]. Additionally, 80 mg/kg daily of chebulanin for 3 weeks significantly suppressed the progression and development of RA in a collagen-induced arthritis mouse model by decreasing the arthritis severity score, attenuating paw swelling and joint destruction, and reducing IL-6 and TNF-*α* amounts, excised phosphorylated (p)-p38 and p-p65, phosphorylated-c-JUN N-terminal kinase, and phosphorylated NF-*κ*B inhibitor alpha, but without effects on extracellular-signal-regulated kinase levels [231].

Punicalagin at 50 mg/kg/d also showed the capacity to prevent the translocation of p-65 and avoid the phosphorylation of IkK and Ik Bα by modulating NF-*κ*B pathway in arthritic rats. It also reduced TNFα, IL-6, CD86, CCR7, CD40, and MHC II expression, raised IL-10 expression, and suppressed dendritic cells migration, which, in turn, diminished the differentiation of Th1, Th17, and Th17/Th1-like and promoted the generation of Tregs via the regulation of dendritic cell maturation [288]. Syringaldehyde at 10, 25, and 50 mg/kg also showed the ability to alleviate paw and joint edema, reduce TNF-*α* and IL-6 levels, and increase the level of IL-10 in arthritic rat serum [289]. Lower levels of IL-6 and TNF-*α* were also obtained by Toy and colleagues at doses of 100 mg/kg and 200 mg/kg/bw [345]. Furthermore, 50 mg/kg *Clitoria ternatea* L. flower petals and 2.5 mg/kg of its major compound, which is quercetin-3ß-D-glucoside, showed the ability to reduce MPO activity and lower pro-inflammatory cytokines, chemokines, reactive oxygen and nitrogen species, and TNFR1, TLR2, iNOS, COX-2, and MMP-2 expression levels in rats with RA [346]. Similar effects were observed with 150 mg/kg Berberis orthobotrys Bien ex Aitch, which also offered protection against arthritic lesions, oxidative damage, and body weight alterations, ameliorated altered hematological parameters, the rheumatoid factor, and contributed to positively modified radiographic and histopathological changes [347]. Furthermore, 250, 500 and 750 mg/kg *Diospyros malabarica* (Desr.) Kostel fruits also showed the capacity to raise anti-inflammatory enzymes and diminish the anti-inflammatory ones in RA rat model [348].

On the other hand, the daily administration of 100 mg/kg *ρ*-coumaric acid for 2 weeks showed remarkable capacity to suppress paw edema and body weight loss and curtail cartel [316], age, bone erosion, TNF-*α*, IL-1*β*, IL-6, IL-17, and MCP-1 quantities in serum and ankle joints of arthritic rats, as well as the expression of RANKL and TRAP, iNOS and COX-2, JNK, p-JNK, and ERK1/2., namely, by regulating RANKL/OPG imbalance and inhibiting RANKL-induced NFATc-1 and c-Fos expression [238]. Similar data were reported by Pragasam and colleagues [349]. Focusing on chlorogenic acid, at 10 mg/kg, it can attenuate liposaccharide-induced bone loss based on micro-computed tomography and histologic analysis of femurs from arthritic rats [315].

Theaflavin-3,3′-digallate administrated at 10 mg/kg three times per week for 9 continuous weeks also showed the capability of reducing the expression of IL-1*β*, TNF-*α*, and IL-6, as well as MMP-1, MMP-2, and MMP-3 amounts in the synovium of a collagen-induced arthritis mouse model, chiefly by inhibiting the activation of NF-*κ*B and the phosphorylation of P38, JNK2, and ERK [230]. Moreover, 50 mg/kg cinnamtannin D1, a polyphenolic compound isolated from *Cinnamomum tamala*, alleviates the severity of RA, affording reduced clinical scores and paw swelling; reduces inflammatory cell infiltration, cartilage damage in the joints, and IL-17, IL-6, and IL-1*β*; enhances TGF-β and IL-10 levels; reduces the frequency of Th17 cells; and enhances the frequency of Treg cells, namely by its potential to inhibit aryl hydrocarbon receptor expression and phospho-STAT3/RORγt [350]. On the other hand, 200 mg/kg cinnamon barks reduce paw volume, weight loss, and, IL-2, IL-4 and IFNγ in RA mice model [351].

N-feruloylserotonin, a natural polyphenol that belongs to indole hydroxycinnamic acid amides, administered at 3 mg/kg/day orally reduced C-reactive protein in plasma and the activity of LOX in the liver rats. It also reduced mRNA transcription of TNF-*α* and iNOS in the liver and IL-1*β* in plasma and IL-1*β* mRNA expression in the liver and spleen of arthritic rats [352].

Moreover, 100 and 200 mg/kg extra virgin olive oil showed the capability to decrease inflammatory markers, joint edema, cell migration, cartilage degradation, and bone erosion in a collagen-induced arthritis model mice, namely, by inhibiting c-Jun N-terminal kinase, p38, signal transducer, and activator of transcription-3, as well as reducing COX-2 and microsomal prostaglandin E synthase-1 expression [353]. Furthermore, 0.05% hydroxytyrosol acetate, a polyphenol present in extra virgin olive oil, showed the ability to diminish IgG1 and IgG2a, COMP, MMP-3, TNF-*α*, IFN-*γ*, IL-1*β*, IL-6, IL-17A, and the MAPKs JAK/STAT and NF-*κ*B pathways and enhance Nrf-2 and heme oxygenase-1 in mice with collagen-induced arthritis [354].

In addition, 50, 100, and 400 mg/kg mangiferin inhibits mRNA expression of cytokine genes in the thymus and spleen of mice with induced-arthritis and lead to decreased serum levels of IL-1*β*, IL-6, TNF-*α*, and RANKL by downregulating NF-*κ*B and activating ERK1/2 [355].

Sarcococca saligna plant at 250 mg/kg also showed ability to diminish IL-1*β*, IL-6, COX-2, prostaglandin E2, TNF-*α* and NF-*κ*B levels, and the arthritic index and paw inflammation and enhance the expression of IL-4 and IL-10 [236]. On the other hand, 10 mg/kg curcumin showed similar effects as the methotrexate (0.5 mg/kg) positive control in reducing TNF-*α* and IL-1*β* in both the synovial fluid and blood serum of arthritic rats [356]. Aqueous extracts of *Dichrostachys cinerea* Wight et Arn. fruits at doses of 150.96 mg GAE/g and 75.48 mg GAE/g reduced IL-1*β*, IL-6, TNF-*α*, and cortisol levels, lipid peroxidation, and NOx production [357]. In addition, 170–1350 mg/kg of the *Circaea mollis* Sieb. and Zucc. plant can also reduce paw and inflammatory swelling, the arthritis index, and TNF-*α* and IL-1*β* and increase the production of serum IL-10 in Freund’s complete adjuvant-induced arthritis model in rats [358]. Opuntia littoralis at 10 and 20 mg/100 g bw also revealed the potential to reduce joint inflammation, paw swelling, edema, MDA, and IL-1*β*, IL-6R, IL-6, IL-17, and IL-23 and ameliorated COX-2, NF-*κ*B, STAT-3, PTEN, and RANKL expression, namely, by upregulating the expression of miR-28 and miR-199a [239]. Furthermore, 25 and 100 mg/kg *Antrocaryon micraster* A. Chev. and Guillaumin seed extract also showed capacity to diminish cachexia, paw edema, infiltration of inflammatory cells, pannus formation, and synovium damage in rats with induced RA [359]. Moreover, the addition of 20% dried plums into the normal diet of transgenic mice with induced-RA showed notable protective effects in protecting articular cartilage, reducing synovitis, IL-1*β*, MCP1, MIP1α, MMP1, and MPP3, and RANKL expression and repressing TNF-induced formation of osteoclasts and mRNA levels of cathepsin K and MMP9 by inhibiting nuclear factor of activated T-cells, cytoplasmic 1 (NFATc1) expression, and NF-*κ*B activation [360]. On the other hand, 750 mg/kg *Opuntia monacantha* showed effectiveness in reducing paw edema, the arthritic score, the rheumatoid factor, inflammation, COX-2, IL-6, TNF-*α*, IL-1, NF-*κ*B, bone erosion, and pannus formation, restoring hemoglobin, white blood count, and platelet parameters, increasing catalase and superoxide dismutase levels, and enhancing IL-4 and IL-10, namely, by its potential to inhibit glutaminase 1 activity in a RA rat model [361]. In addition, 800 mg/kg Solanum nigrum also showed effectiveness in reducing paw edem, and restoring body weight, hematologic parameters, and radiographic and histopathologic alterations towards normal in different RA rat models [362].

In addition, 15 mg/kg quercetin and 10 and 20 mg/kg quercetin-loaded chitosan showed the capacity to reduce TNF-*α* and IL-6, as it was observed that the nanoencapsulation of quercetin is an added value to improve its efficacy in rats with induced RA [363]. In another study, 1.25 g/kg grape polyphenols mixed with 1.25 g/kg propolis showed potential to diminish the intensity of cachexia and alleviate RA scores in RA female rats [364]. Reduced cachexia and arthritic paw scores were also reported by Decendit [150], who treated chronic rat adjuvant-induced arthritis with 125 mg/kg malvidin 3-*O*-*β* glucoside.

*Phoenix dactylifera* L. seeds can diminish IL-1*β* levels, paw edema, the erythrocyte sedimentation rate, and C-reactive protein in rats [365].

Finally, the liposomal drug delivery system for morin, a dietary polyphenol, showed the capacity to ameliorate RA in rats, mainly by increasing its uptake by synovial and spleen macrophages, reducing mRNA expression and consequent production of TNF-*α*, IL-1*β*, IL-6, IL-17, RANKL, STAT-3, p-STAT-3, VEGF, iNOS, and NF-*κ*B-p65, and increasing the expression of osteoprotegerin [235].

##### Clinical Trials

Regarding clinical trials, it was found that 500 mL/day of low-calorie cranberry juice is able to attenuate RA symptoms in women with this disease after a 90-day treatment by decreasing anti-cyclic citrullinated peptide antibodies levels, pain intensity, and swollen joints; however, no inflammatory biomarkers were verified [366]. Additionally, the daily ingestion of 500 mL/day of low-calorie cranberry juice with 3 g of fish oil *ω*-3 fatty acids showed effectiveness in C-reactive protein, the erythrocyte sedimentation rate, and related-pain [367]. On the other hand, pomegranate extract (250 mg for 8 weeks), in addition to alleviating swollen, pain intensity, and tender joints in patients with, can also diminish the erythrocyte sedimentation rate and morning stiffness and enhance glutathione peroxidase. No alterations were found in CRP, MDA, or MMP3 levels [368]. Finally, a 1 g daily dose of resveratrol for 3 months [369] also showed effectiveness in reducing joint swelling and tenderness, as well as TNF-*α*, IL-6, protein C-reactive, MMP-3, the erythrocyte sedimentation rate, and undercarboxylated osteocalcin in patients with RA of both genders [369].

## 6. Conclusions

In general, the present review provides evidence that supports the therapeutic effect of polyphenols. Polyphenols are largley present in nature and seem to be a promising approach to the boost immune system and contribute to a healthy status. Contrary to chemical pharmaceutics that originate several side effects and their loss of efficacy over time, it is largely belief that phenolics are more effective and a safer option. This is mainly due to their capacity to counteract oxidative stress and interact with inflammatory pathways. In this way, it is not surprising that their use as an adjuvant to conventional antirheumatic drugs is considered an added value. However, although phenolics are easy to obtain and economical, most of them present poor bioavailability and stability and rapid metabolism. Therefore, their encapsulation is beneficial to increase their efficacy. In addition, their safety profile and clinical trials need to be more explored.
